# A Chemical Biology Approach to Understanding Molecular Recognition of Lipid II by Nisin(1–12): Synthesis and NMR Ensemble Analysis of Nisin(1–12) and Analogues

**DOI:** 10.1002/chem.201902814

**Published:** 2019-10-10

**Authors:** Rachael Dickman, Emma Danelius, Serena A. Mitchell, D. Flemming Hansen, Máté Erdélyi, Alethea B. Tabor

**Affiliations:** ^1^ Department of Chemistry University College London, 20 Gordon Street London WC1H 0AJ UK; ^2^ The Swedish NMR Centre Medicinaregatan 5 40530 Gothenburg Sweden; ^3^ Department of Chemistry–BMC Uppsala University Box 576 75123 Uppsala Sweden; ^4^ Institute of Structural and Molecular Biology Division of Biosciences University College London Gower Street London WC1E 6BT UK

**Keywords:** antibiotics, cyclic peptides, lantibiotics, NMR spectroscopy, solid phase synthesis

## Abstract

Natural products that target lipid II, such as the lantibiotic nisin, are strategically important in the development of new antibacterial agents to combat the rise of antimicrobial resistance. Understanding the structural factors that govern the highly selective molecular recognition of lipid II by the N‐terminal region of nisin, nisin(1–12), is a crucial step in exploiting the potential of such compounds. In order to elucidate the relationships between amino acid sequence and conformation of this bicyclic peptide fragment, we have used solid‐phase peptide synthesis to prepare two novel analogues of nisin(1–12) in which the dehydro residues have been replaced. We have carried out an NMR ensemble analysis of one of these analogues and of the wild‐type nisin(1–12) peptide in order to compare the conformations of these two bicyclic peptides. Our analysis has shown the effects of residue mutation on ring conformation. We have also demonstrated that the individual rings of nisin(1–12) are pre‐organised to an extent for binding to the pyrophosphate group of lipid II, with a high degree of flexibility exhibited in the central amide bond joining the two rings.

## Graphical abstract

**Lipid recognition**: To probe the structural factors governing the highly selective molecular recognition of lipid II by the N‐terminal region of nisin, analogues of nisin(1‐12) were synthesised. NMR ensemble analysis of analogue and wild‐type structures reveals the favourable conformations available to these bicyclic ring structures.

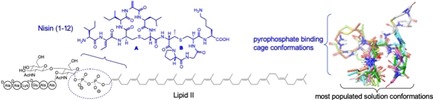

## Introduction

**Figure 1:**
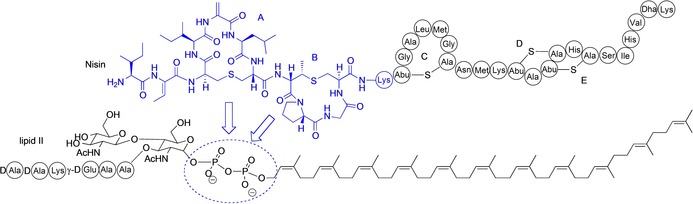
Interactions between the N‐terminus of nisin and the pyrophosphate group of lipid II.

Antibiotic resistant infections are becoming an increasing threat to global public health,^1^ which has generated a renewed interest in natural products as a source of potent antimicrobial drugs. One such class of compounds is the lantibiotics: a family of gene‐encoded antimicrobial peptides which are extensively post‐translationally modified. The lantibiotics have complex cyclic structures generated by the thioether‐bridged amino acids lanthionine (Lan) and methyllanthionine (MeLan), and frequently also contain the α,β‐unsaturated amino acids dehydroalanine (Dha) and dehydrobutyrine (Dhb).^2^, ^3^ Nisin (Figure 1), the most commonly studied lantibiotic, is used commercially as a food preservative,^4^ and has broad‐spectrum activity against a range of Gram‐positive organisms, including methicillin‐resistant *Staphylococcus aureus* (MRSA).^5^ The mechanism of action of the lantibiotics is mediated by high affinity binding to lipid II, a key intermediate in peptidoglycan biosynthesis.^6^ In the case of nisin, this interaction results in the rapid formation of stable pores in the bacterial membrane, of which lipid II is an intrinsic component, at an 8:4 nisin:lipid II ratio.^7^ A second effect of this binding is the inhibition of peptidoglycan biosynthesis, caused by the large‐scale sequestration and aggregation of lipid II.^8^, ^9^ The importance of the interaction with lipid II in the antibacterial action of nisin has also been demonstrated in functional studies, in which it was shown that nisin(1–12) is able to antagonize the activity of WT nisin.^10^

NMR studies have proved to be a valuable method in the study of lantibiotic conformation and lipid II binding.^11^ Indeed, the solution conformation of nisin^12^, ^13^, ^14^ and a number of other lantibiotics, such as subtilin,^15^ mutacin 1140^16^ and cinnamycin,^17^ have been reported. The interaction between nisin and lipid II has also been investigated using NMR. Initially, studies were conducted with nisin in lipid II‐doped micelles, revealing that the N‐terminal of nisin is involved in target recognition and binding to the lipid.^18^, ^19^ An NMR study in DMSO with full length nisin and a truncated analogue of lipid II at a 1:1 ratio later revealed the nature of the interaction, which involves the binding of the nisin A and B rings (residues 1–12) in a cage‐like formation around the pyrophosphate of lipid II (PDB ID 1WCO).^20^ Isothermal calorimetry (ITC) and vesicle leakage studies have also confirmed the importance of the pyrophosphate group for nisin binding, and have demonstrated that the MurNAc moiety is required for high affinity interaction.^21^ A similar binding mode has also been observed in NMR studies of the two‐component lantibiotic lacticin 3147, in which the C‐terminus of the A1 peptide forms a cage around the lipid II pyrophosphate.^22^ Recently, Weingarth et al. reported the solid state NMR of lipid II‐bound nisin as part of the pore complex in DOPC liposomes and in native *Micrococcus flavus* membranes.^23^ Although this study confirmed the broad features of the 8:4 pore complex, the authors observed that the spectra under these conditions differed drastically from that of the previously reported spectra of lipid II‐bound nisin in DMSO, suggesting that nisin adopts a different conformation in the native pore.^20^

In addition to structures of full‐length lantibiotics, the conformations of the A and B rings of nisin have also been investigated. For example, Palmer et al. reported the solution structures of analogues of nisin ring A and ring B,^24^ which adopt conformations remarkably similar to those found in the full length wild‐type (WT) peptide in aqueous solution.^14^ Recently, we have also studied^25^ the conformations of the individual A and B rings of nisin and another class I lantibiotic, mutacin I.^26^ Although potentially useful for determining to what extent the isolated lantibiotic rings are pre‐organized for lipid II binding, one disadvantage of such studies is that they provide no insight into how each ring may affect the conformation of the other, or on the relative orientation of the two rings. Given that flexibility in the nisin hinge region is essential for bioactivity,^6^ and that large changes of torsion angle between lantibiotic rings are important to enable mersacidin‐lipid II binding,^27^ it is perhaps surprising that a conformational study of the entire Ring A–Ring B structure, nisin(1–12), has not yet been conducted.

Another factor to consider, especially in the interest of developing more stable antibiotics based on the structure of nisin,^28^, ^29^, ^30^, ^31^ is the effect of residue mutation within lantibiotic binding rings on either solution conformation or antibacterial activity. Significant efforts have been directed towards understanding the effects of dehydro residue replacement, as these residues contribute to the metabolic instability of these peptides, however no clear picture has yet emerged. Palmer et al. have shown that substitution of Dha5 for Ala in nisin ring A leads to significant conformational change of the isolated ring A,^32^ conversely, our NMR studies^25^ comparing isolated ring A structures of nisin and mutacin I with saturated analogues of mutacin I ring A indicated that the replacement of Dha5 by either Ser or Ala did not significantly affect the overall conformation of the Leu4‐Xaa5‐Leu6 portion of ring A. This is supported by mutation studies. The observation that full length nisin bearing a Dha5Ala mutation retains bioactivity against *Micrococcus luteus* 33 suggests that any conformational change caused by dehydro replacement does not affect the activity of nisin, and therefore that it may not interfere with lipid II binding. Similarly, Wiedemann et al. have shown that the replacement of Dhb2 in full length nisin with either Ser, Ala or Val has little effect on the MIC.^6^ Other groups have investigated the effect of (Me)Lan replacement. Slootweg et al. synthesized dicarba bridged analogues of nisin(1–12) by RCM, finding that replacement of (Me)Lan with longer dicarba bridges was reasonably well tolerated.^34^ As part of this work the authors also investigated the effect of replacing both Dha and Dhb with Ala, and found that the presence of the dehydro residues increased the affinity of the dicarba bridged peptides for lipid II. Introduction of a third cyclic constraint in dicarba bridged analogues of nisin(1–12), by creating a lactam bridge between the N‐terminus and the B ring, has also been investigated by Harmsen et al.^35^ The resulting reduction in flexibility increased the affinity of the peptide for lipid II over the bicyclic dicarba analogue, but was still five‐fold less active than WT nisin(1–12).

Flexible molecules, such as nisin(1–12) and the analogues described above, exist in a number of rapidly equilibrating conformations in solution. Experimental NMR variables, such as NOEs and *J*‐couplings, are therefore averaged over the whole population of solution conformations, and a single average structure can be an inadequate representation of the true conformations present in solution.^36^ A good method for the study of the conformations of flexible molecules is NAMFIS (NMR analysis of molecular flexibility in solution).^37^ In the NAMFIS technique, the averaged NMR variables are deconvoluted by varying the molar fractions of a computational theoretical ensemble, calculated by unrestrained Monte Carlo molecular mechanics, until the best possible fit of the experimental NMR data is obtained. The result of this is an ensemble of all conformations which are present in solution, and their probabilities, hence providing a more complete picture of the shape of the compound in solution than is possible by average structure calculation. The utility of NAMFIS analysis in determining the solution conformation of small cyclic peptides has been demonstrated,^38^, ^39^, ^40^ but has never previously been applied to lantibiotic systems.

The first aim of this research was to further develop our existing solid‐phase peptide synthesis (SPPS) methodology to enable the synthesis of two analogues of nisin(1–12) bearing dehydro residue replacements and a MeLan→Lan substitution. Our second aim was to examine in detail the solution conformations of these peptides using the NAMFIS method and compare them to the conformations of WT nisin(1–12) in order to explore the effect of residue mutation on ring conformation and pre‐organization for lipid II binding. Our third aim was to carry out a detailed conformational analysis of WT nisin(1–12) itself, in order to elucidate further how this peptide sequence achieves highly selective binding to lipid II, and to compare the conformations to the previously published structures of full length nisin, both alone and bound to lipid II.

## Results and Discussion

### Peptide synthesis

**Figure 2:**
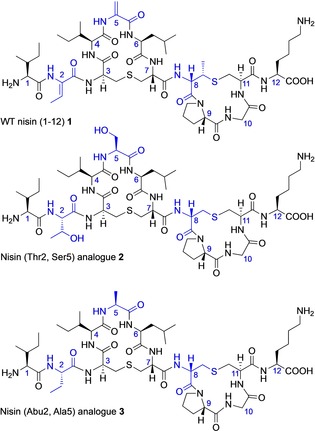
Structures of ring AB peptides. Positions of modification are highlighted in blue.

Several different approaches to the chemical synthesis of lantibiotics have been reported over the past 20 years.^41^, ^42^ We have developed very effective solid‐phase peptide synthesis methodology which we and others have applied to the synthesis of individual rings of lantibiotics,^43^, ^44^ overlapping rings^45^ and to the total synthesis of complete lantibiotics.^46^, ^47^, ^48^, ^49^ We have previously reported the solid‐phase synthesis of the individual A and B rings of nisin and of the related lantibiotic, mutacin I,^25^ and have investigated the conformational properties of these isolated rings and synthetic analogues by NMR. We have now extended this work to prepare synthetic analogues of WT nisin(1–12), which can be compared with WT nisin(1–12) itself (**1**) (Figure 2). (Thr2, Ser5) analogue **2** was designed using the amino acids in the 2‐ and 5‐positions that would be present in the biosynthetic precursor peptide, and that would undergo dehydration by the enzyme NisB in the producing organism.^50^ Similarly, to investigate the effects of dehydro amino acids on the conformation of the bicyclic structure, (Abu2, Ala5) analogue **3** was designed with the saturated analogues of Dhb and Dha in positions 2 and 5 respectively. We envisaged that these chemically modified nisin analogues would be more stable than the parent nisin structure, and for analogue **2** we also expected that the Thr and Ser residues would also offer some improvement in aqueous solubility of the resulting peptide. In both analogues **2** and **3**, we also substituted Lan for the naturally‐occurring MeLan in ring B, as our previous studies had indicated that replacement of Lan for MeLan did not significantly change the backbone conformation.^25^

**Scheme 1:**
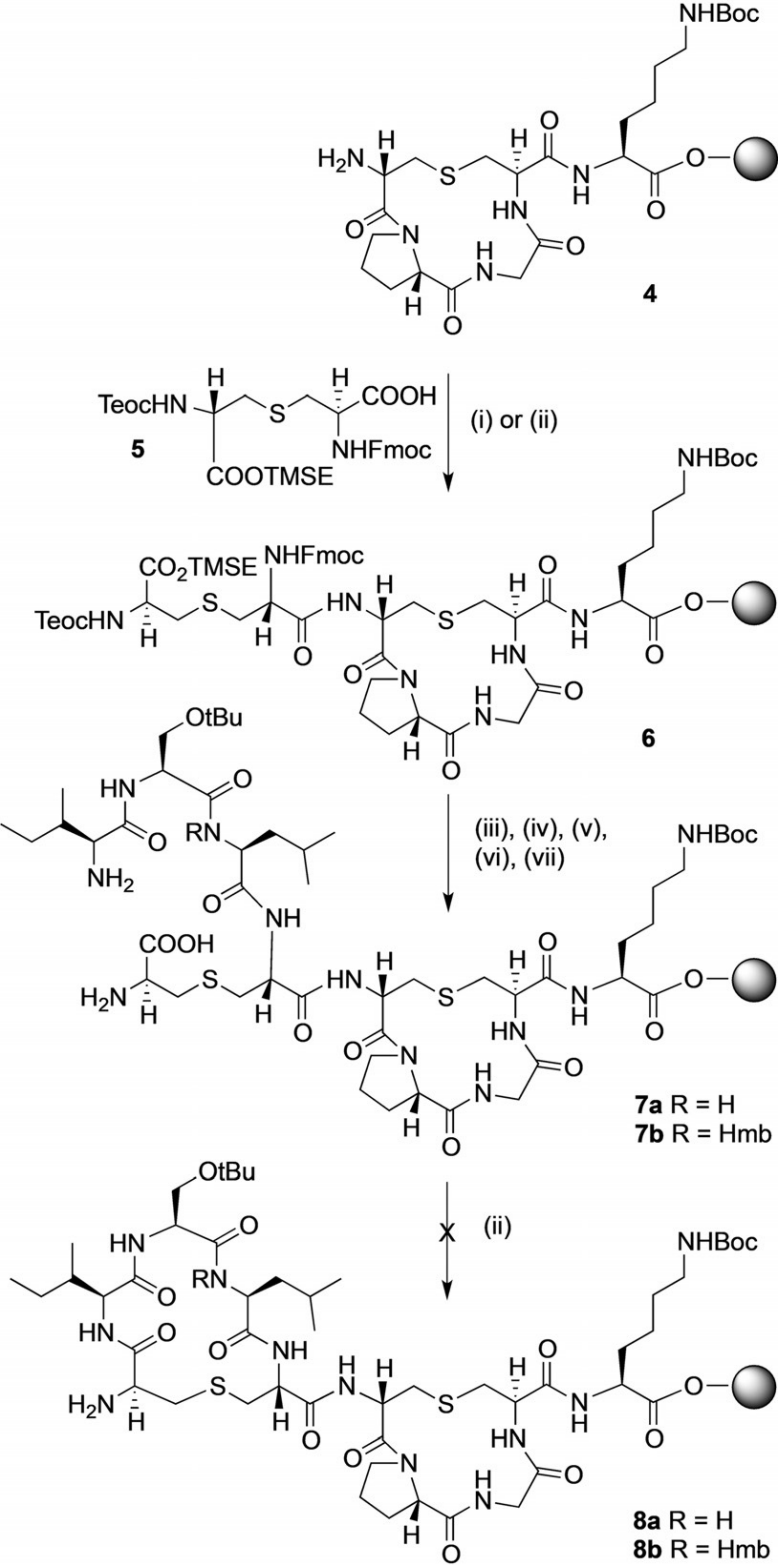
Attempted SPPS of (Thr2, Ser5) analogue **2**. Reagents and conditions: (i) HOAt, PyAOP, DIPEA, DMF; (ii) HOAt, PyAOP, DIPEA, DMF, μwave 60 °C for 5 min; (iii) Fmoc deprotection (piperidine, DMF) followed by coupling (Fmoc‐Leu‐OH, HOAt, PyAOP, DIPEA, DMF)*×*2 (**7 a**) or coupling Fmoc‐(FmocHmb)Leu‐OH, HBTU, DIPEA, DMF)*×*2 (**7 b**); (iv) Fmoc deprotection (piperidine, DMF) followed by coupling (Fmoc‐Ser(*t*Bu)‐OH, HOAt, PyAOP, DIPEA, DMF)*×*2; (v) Fmoc deprotection (piperidine, DMF) followed by coupling (Fmoc‐Ile‐OH, HOAt, PyAOP, DIPEA, DMF)*×*2; (vi) Teoc/TMSE deprotection (TBAF, DMF); (vii) Fmoc deprotection (piperidine, DMF).

Our route to (Thr2, Ser5) analogue (**2**) started from the previously reported^25^ resin‐bound cyclic ring B peptide **4**. We initially attempted to couple the next (Teoc, TMSE/Fmoc) Lan monomer **5** 45 to the free ‐NH_2_ group of **4** to give resin‐bound intermediate **6**, and then to build up the linear precursor to ring A, using standard Fmoc SPPS coupling conditions (HOAt and PyAOP) (Scheme 1). However, only trace amounts of product with extensive impurities were obtained. Mass spectrometry analysis indicated that the initial coupling of the (Teoc, TMSE/Fmoc) Lan monomer **5** was unsuccessful under these conditions. We have previously found^51^ that microwave conditions improve the coupling of orthogonally protected lanthionines to resin‐bound intermediates, and repeating the synthesis using microwave coupling for the incorporation of **5** was successful. Subsequent addition of the remaining amino acids in the sequence of ring A, removal of the Fmoc group and selective Teoc and TMSE deprotection gave the resin‐bound intermediate **7 a**. However, all attempts to cyclise **7 a** on‐resin, using standard coupling conditions, were unsuccessful. Mass spectrometry of intermediate peptides cleaved from the resin showed only trace amounts of peptide **8 a** had been formed (Supporting Information, Figure S1).

We hypothesized that the resin‐bound intermediate **7 a** was folded on‐resin in a conformation where the amino group of Ile4 was remote from the carboxylic acid group of Lan3. Techniques to improve the synthesis of “difficult peptides” have been extensively researched.^52^ Such peptides contain sequences which show a high tendency to fold or aggregate on‐resin, masking the nascent amino group and resulting in low or failed peptide coupling steps. Many of the approaches used to overcome these problems focus on preventing the formation of inter‐ or intra‐chain hydrogen bonds by masking the amide NH. We reasoned that similar approaches could be used to overcome the failure of **7 a** to cyclise, by diminishing its ability to fold into a non‐productive conformation. We first attempted to improve the on‐resin cyclisation by incorporation of a Hmb‐protected Leu residue (Scheme 1). Hmb‐protected amino acids have previously been used to improve the cyclisation of pentapeptides^53^ and of larger lanthionine‐containing rings.^51^ Although we were able to successfully incorporate Fmoc(Hmb)Leu‐OH to give resin‐bound intermediate **7 b**, chain extension and on‐resin cyclisation did not give the required **8 b**.

Another approach to this problem is the use of pseudoprolines, in which threonine, serine (or cysteine)‐derived dipeptides are protected with proline‐like oxazolidines (or thioazolidines). Many of these dipeptides are commercially available and can be incorporated directly into peptide synthesis protocols. The incorporation of pseudoproline residues into linear sequences has been reported to improve the head‐to‐tail cyclisation of both short^54^ and longer^55^ peptides. This effect was attributed to the observation that such residues induce a predominantly *cisoid* conformation about the amide bond adjacent to the modified amino acid, resulting in the temporary induction of a β‐turn. The presence of a Ser residue in ring A made this an attractive approach to attempt to pre‐organise the peptide for ring closure.

**Scheme 2:**
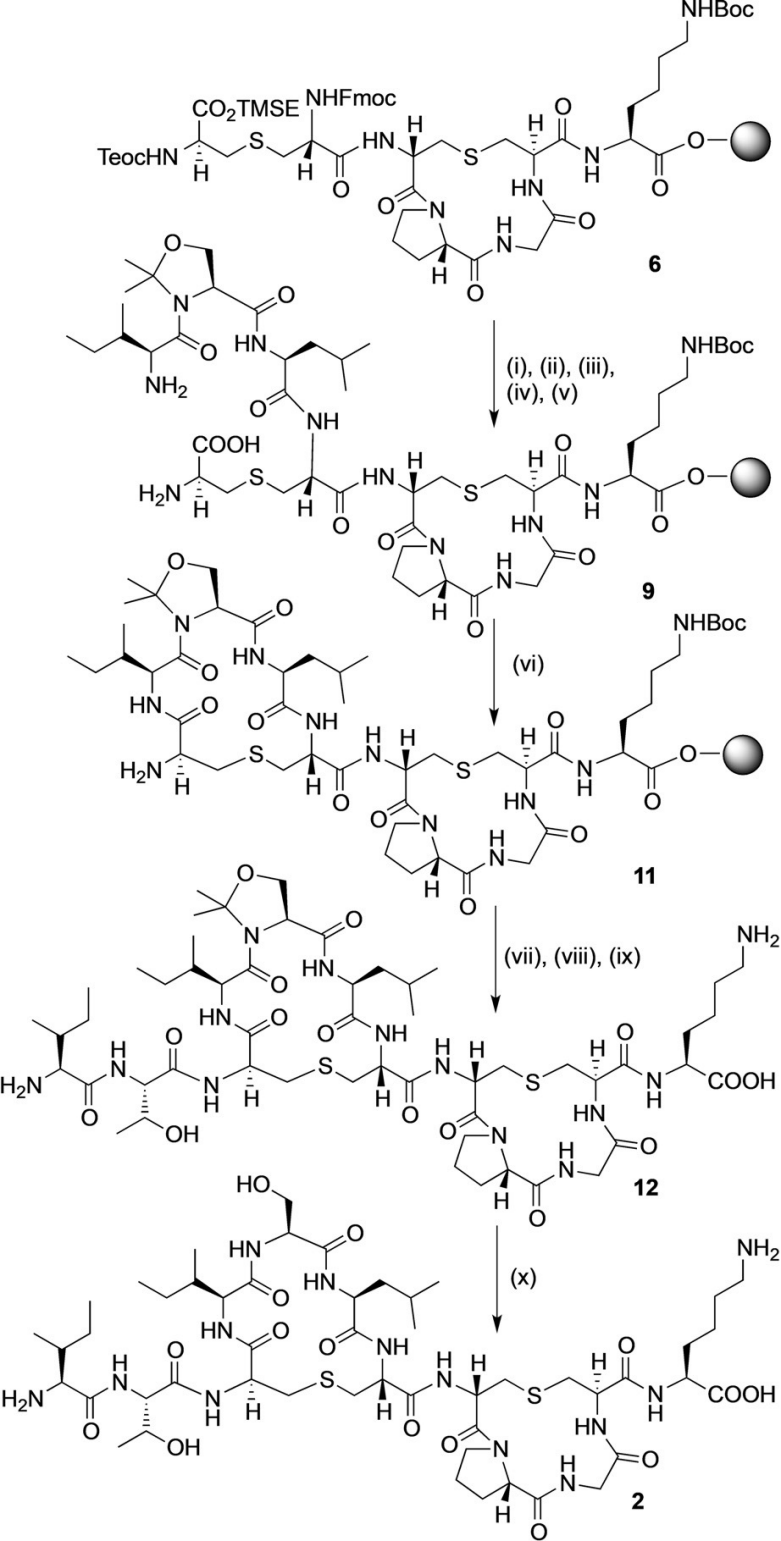
SPPS of (Thr2, Ser5) analogue **2** using a pseudoproline strategy. Reagents and conditions: (i) Fmoc deprotection (piperidine, DMF) followed by coupling (Fmoc‐Leu‐OH, HOAt, PyAOP, DIPEA, DMF)*×*2; (ii) Fmoc deprotection (piperidine, DMF) followed by coupling (Fmoc‐Ile‐Ser[ψ(Me,Me)pro]‐OH, HOAt, PyAOP, DIPEA, DMF)*×*2; (iii) Teoc/TMSE deprotection (TBAF, DMF); (iv) Fmoc deprotection (piperidine, DMF); (v) HOAt, PyAOP, DIPEA, DMF, μwave 60 °C for 5 min; (vi) (Fmoc‐Thr(*t*Bu)‐OH, HOAt, PyAOP, DIPEA, DMF)*×*2; (vii) Fmoc deprotection (piperidine, DMF) followed by coupling (Fmoc‐Ile‐OH, HOAt, PyAOP, DIPEA, DMF)*×*2; (viii) Fmoc deprotection (piperidine, DMF); (ix) TFA, TIPS, H_2_O; (x) triflic acid, 0 °C, 5 min.

We therefore synthesised the resin‐bound intermediate **9**, incorporating the commercially available dipeptide Fmoc‐Ile‐Ser[ψ(Me,Me)pro]‐OH **10** using standard coupling conditions (Scheme 2). Pleasingly, it was then possible to cyclise **9** to give **11**, using microwave coupling conditions. The peptide sequence was completed by the coupling of the two N‐terminal residues, Fmoc‐Thr(*t*Bu)‐OH and Fmoc‐Ile‐OH. Unexpectedly, cleavage from the resin with TFA did not result in acidolysis of the pseudoproline, and the partially deprotected **12** was recovered. Other groups^56^, ^57^, ^58^ have also reported that these proline‐like oxazolidines and thioazolidines were resistant to deprotection with TFA. Subsequent treatment of **12** with TMFSA^56^ at 0 °C gave the desired (Thr2, Ser5) analogue **2** in 3 % overall yield after purification.

**Scheme 3:**
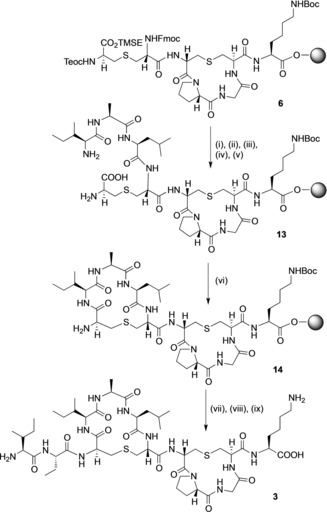
Synthesis of (Abu2, Ala5) analogue **3**. Reagents and conditions: (i) Fmoc deprotection (piperidine, DMF) followed by coupling (Fmoc‐Leu‐OH, HOAt, PyAOP, DIPEA, DMF)×2; (ii) Fmoc deprotection (piperidine, DMF) followed by coupling (Fmoc‐Ala‐OH, HOAt, PyAOP, DIPEA, DMF)*×*2; (iii) Fmoc deprotection (piperidine, DMF) followed by coupling (Fmoc‐Ile‐OH, HOAt, PyAOP, DIPEA, DMF)×2; (iv) Teoc/TMSE deprotection (TBAF, DMF); (v) Fmoc deprotection (piperidine, DMF); (vi) HOAt, PyAOP, DIPEA, DMF, μwave 60 °C for 5 min; (vii) (Fmoc‐Abu‐OH, HOAt, PyAOP, DIPEA, DMF)×2; (viii) Fmoc deprotection (piperidine, DMF) followed by coupling (Fmoc‐Ile‐OH, HOAt, PyAOP, DIPEA, DMF)×2; (ix) Fmoc deprotection (piperidine, DMF) then TFA, TIPS, H_2_O.

The synthesis of (Abu2, Ala5) analogue **3** was also carried out from the resin‐bound intermediate **6** (Scheme 3). The three ring A residues, Leu, Ala and Ile were added by standard SPPS methods and the Teoc, TMSE and Fmoc groups removed to give **13**. Cyclisation was carried out on‐resin to give the resin‐bound bicyclic peptide **14**. This was followed by chain extension with Fmoc‐Abu‐OH and Fmoc‐Ile‐OH, and cleavage from the resin, to give analogue **3**. Using this synthetic protocol, the purity of the bicyclic analogue was poor and after extensive purification the peptide was isolated in 0.5 % yield, insufficient to allow full structural assignment. As with analogue **2**, the poor yield and purity may also be attributable to the failure of the resin‐bound intermediate **13** to fold into a conformation where cyclisation is possible. Unfortunately, a pseudo‐proline approach is not possible with the three ring A residues present in analogue **3**. We attempted to improve the yield of **3** by incorporation of either Fmoc(Hmb)Leu‐OH or Fmoc(Hmb)Ala‐OH, as appropriate, but none of the desired bicyclic peptide could be isolated using this approach.

Digestion of commercially available nisin to give WT nisin(1–12) **1** has been extensively described in the literature, and has been used by a number of groups to produce fragments of the WT peptide for various applications. As commercially available nisin from *L. lactis* contains only approximately 2.5 % nisin, prior enrichment and removal of salts and denatured milk solids is required. We initially followed the enrichment method reported by Slootweg et al.,^59^ giving pure **1** for structural analysis. However, the modified digestion procedure described by Koopmans et al.,^28^ in which higher concentrations of nisin and trypsin were used, was found to require shorter reaction times, hence decreasing the risk of lanthionine oxidation caused by extended periods of incubation in buffer.

### NAMFIS analysis

WT nisin(1–12) (**1**) and (Thr2, Ser5) analogue (**2**) were characterised by NMR in [D_6_]DMSO, with structural assignments derived from COSY, TOCSY, NOESY, HSQC and HMBC NMR spectra. NOESY spectra were recorded with mixing times of 100, 200, 300, 400, 500, 600 and 700 ms without solvent suppression. Some of the (Thr2, Ser5) analogue (**2**) ring A protons, particularly in Leu6 and Lan7, were not observed in the ^1^H NMR spectra, possibly due to line broadening originating from increased flexibility caused by the Dha5Ser substitution. The initial rate approximation was used to calculate interproton distances for **1** and **2** in the linear build‐up range, using geminal methylene protons as an internal standard (1.78 Å). A total of 44 NOE correlations were observed for nisin(1–12) (**1**), and 34 for (Thr2, Ser5) analogue (**2**). No intraresidue restraints were used in the NAMFIS calculations; however, leaving 16 NOE‐derived distances for nisin(1–12) (**1**) and 15 for (Thr2, Ser5) analogue (**2**) (Tables S1, S2 and Figures S2 and S3). 14 of the 15 correlations for (Thr2, Ser5) analogue (**2**) described distances within ring B, with only 1 correlation describing ring A, though all of the correlations in the NOESY spectra could be attributed to assigned peaks. ^3^ *J*_HA‐HN_ coupling constants were measured for both peptides from ^1^H NMR spectra, from which the backbone dihedral angles were derived via a Karplus equation optimized for peptides.^60^ These were included in the NAMFIS ensemble calculation.

Theoretical ensembles for both peptides were generated using Monte Carlo conformational searches, using two different force fields, followed by molecular mechanics minimization (MCMM) (Table S3). The lipid II‐bound conformation available in the PDB (PDB ID 1WCO)^20^, ^61^ was added to the ensemble for nisin(1–12) (**1**). By deconvolution of the population averaged experimental constraints into the probability‐weighted sum of the back‐calculated constraints from the computational predicted ensembles, we estimated the molar fraction of each theoretical conformer present in solution using the NAMFIS algorithm (Tables S5, S6). The ensemble analyses were validated, following the previous literature,^39^, ^40^ through evaluation of the reliability of the conformational restraints by the addition of 10 % random noise to the experimental data as well as by the random removal of individual restraints.

### Solution conformations of nisin(1–12) (1) and (Thr2, Ser5) analogue (2)

**Figure 3:**
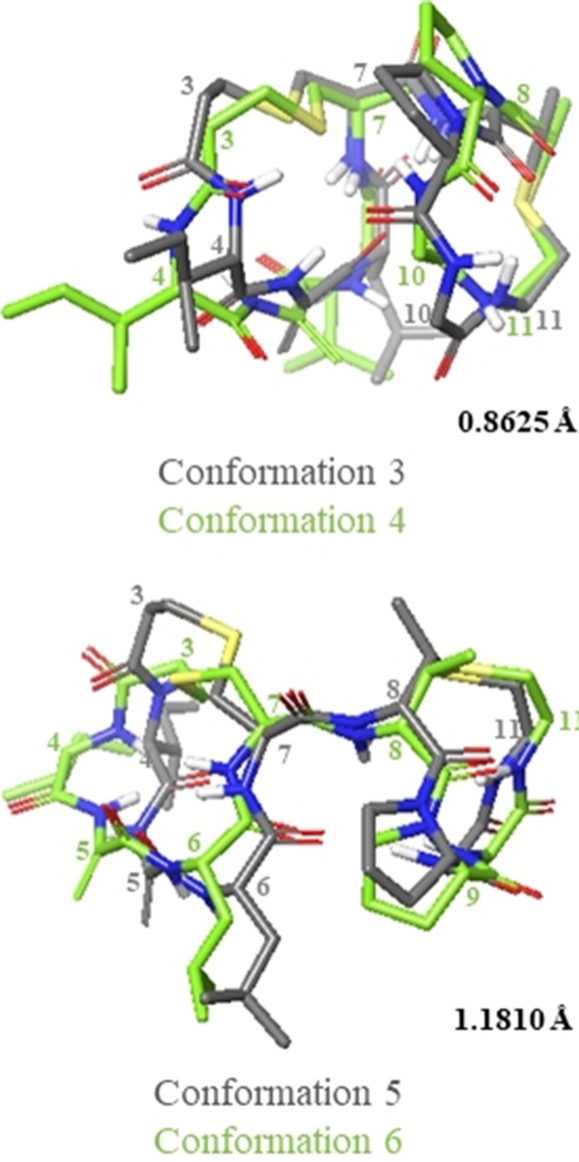
Pairs of nisin(1–12) (**1**) conformations, illustrating the similarity in overall folding between 3 and 4, and between 5 and 6. Ensembles of the lowest energy structures were produced in Maestro (Version 11.4, Schrödinger, LLC), by alignment of αH and S atoms within the lantibiotic rings.

**Figure 4:**
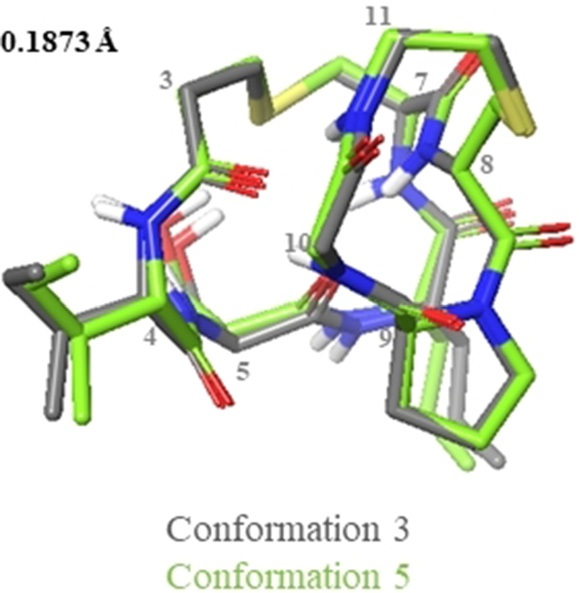
Pair of (Thr2, Ser5) analogue (**2**) conformations showing the similarity of orientation between the A and B rings.

NAMFIS analysis of the nisin(1–12) (**1**) ensemble revealed that the peptide exists in a total of 6 conformations in solution (Figure S4, Table S4). Of these 6 conformations, we can distinguish two pairs of conformers which exhibit similar folds (Figure 3). One of these pairs (conformations 3 and 4) make up the most populated solution conformations, with a combined population of 52 %. The second pair (conformations 5 and 6, where 6 is the structure of lipid II‐bound nisin taken from the PDB, ID 1WCO^20^, ^61^) correspond to the lipid II‐bound conformation. This indicates that in DMSO solution, the population of the peptide adopting the lipid II‐bound conformation is approximately 26 %. NAMFIS analysis of the (Thr2, Ser5) analogue (**2**) ensemble showed that the peptide exists in a total of 5 conformations in solution (Figure S5, Table S4), though the backbone structure within the rings is almost identical in conformers 3 and 5 (Figure 4).

These conformations are also the highest molar fraction, with a combined population of 49 %. Among the conformations of each peptide selected by NAMFIS analysis, there is a high degree of similarity within each of the rings, that is, either ring A or ring B can be aligned with low RMSD. However, there is a high degree of flexibility around the central amide bond between the two rings, leading to a set of structures with diverse overall backbone conformation and high global RMSD (Table 1).

**Table 1:** Local and global RMSDs of nisin(1–12) and Thr/Ser analogue aligned to ring A and ring B.

		Max. RMSD in Å	
		Local	Global
Nisin(1–12)	aligned to ring A	1.1248	7.3754
	aligned to ring B	0.5184	9.4137
Thr/Ser analogue	aligned to ring A	1.3395	3.9736
	aligned to ring B	1.0325	8.8228

**Figure 5:**
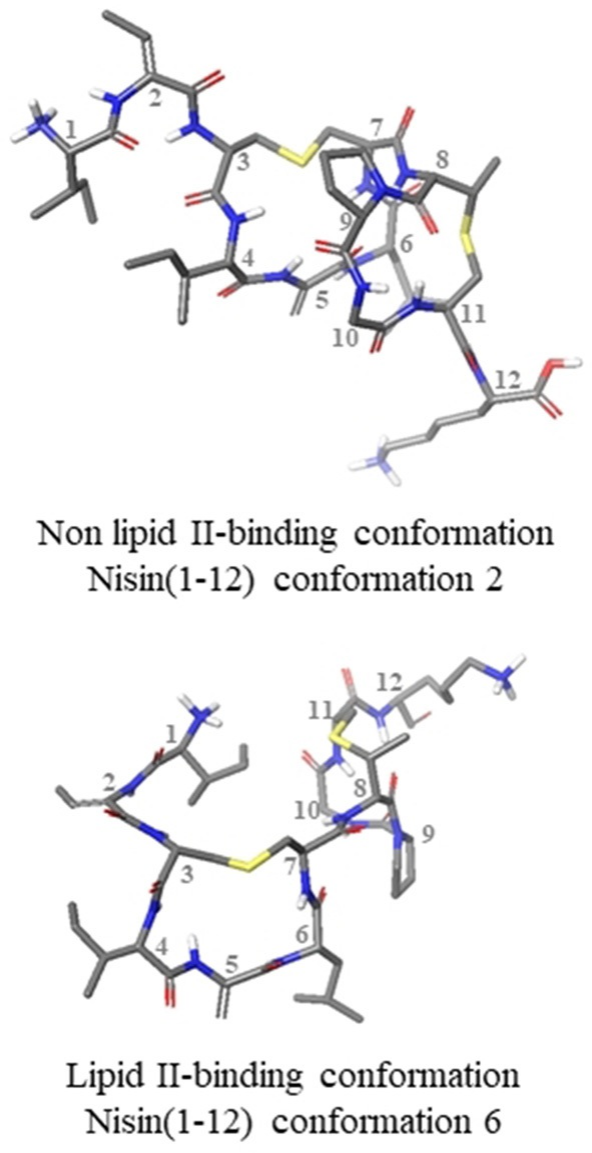
Examples of lipid II bound and unbound conformations adopted by nisin(1–12). Comparisons of all conformations in each NAMFIS solution are in Figure S6.

**Figure 6:**
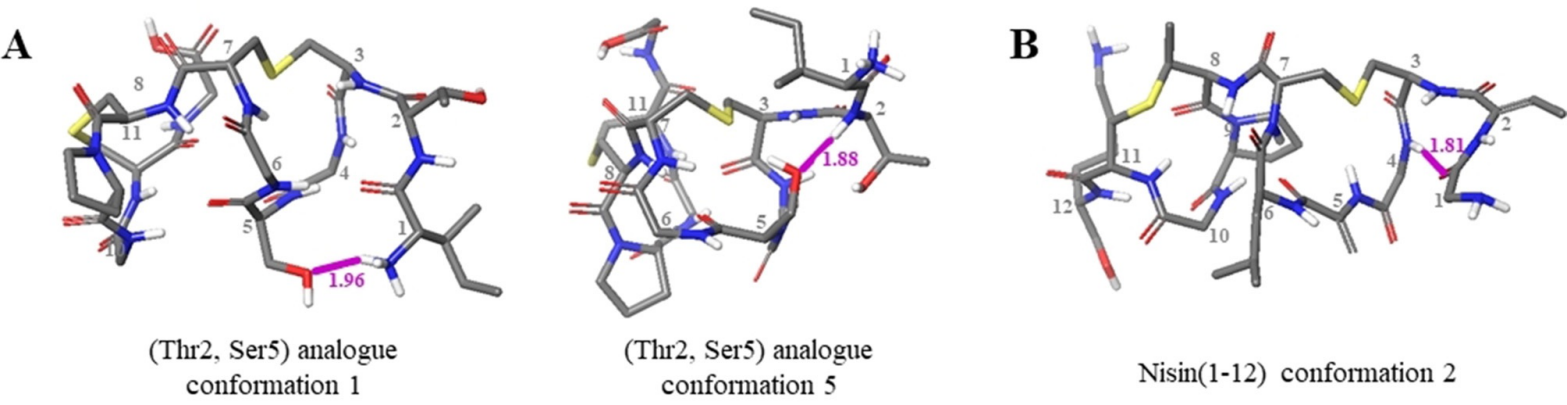
Positions of predicted hydrogen bonds in selected conformations of nisin(1–12) and (Thr2, Ser5) analogue. Hydrogen bonds are indicated by pink lines.

In the case of nisin(1–12) (**1**) there is a large range of rotation, enabling the peptide to adopt the lipid II‐bound conformation, whereas in the (Thr2, Ser5) analogue (**2**), each conformation has the same overall fold (relative to ring A, the B rings all rotate in the same direction) and do not adopt lipid II‐bound conformations (Figure 5). Examination of the (Thr2, Ser5) (**2**) analogue conformations revealed that this difference may be due to the presence of a hydrogen bond between the Ser OH and either Ile1 or Thr2 in all cases. In conformations 1–4, the hydrogen bond is to the terminal amine in Ile1, and in conformation 5 it is to the Thr NH (Figure 6 A). This hydrogen bond appears to fix the first two residues over one face of ring A, possibly hindering ring B from adopting the lipid II‐binding conformation. This hypothesis is supported by the observation that a similar ‘blocking“ of one face of ring A is also observed in half of the nisin(1–12) (**1**) conformations (conformations 1, 2, and 4), caused by a hydrogen bond between Ile1 CO and Ile4 NH, resulting in an overall fold similar to that adopted by the (Thr2, Ser5) analogue (**2**) conformations (Figure 6 B).

**Figure 7:**
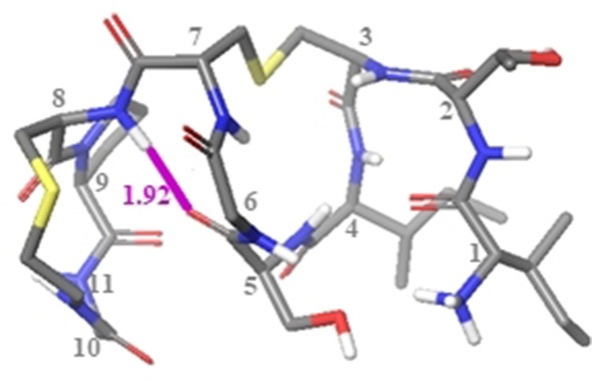
Example of the hydrogen bond formed between residue 5 CO and residue 8 NH in (Thr2, Ser5) analogue conformation 4. The hydrogen bond is indicated by a pink line.

An alternative, or perhaps complementary, explanation for the tendency of the rings to fold as observed in the (Thr2, Ser5) analogue (**2**) conformations is that one or more hydrogen bond(s) can be formed between ring A and ring B which stabilise this orientation. All of the conformations of both nisin(1–12) (**1**) and (Thr2, Ser5) analogue (**2**) which do not exhibit a lipid II‐binding fold contain at least one such hydrogen bond, with the most commonly formed bond being between the carbonyl of residue 5 and the NH of either residue 8 or 10 (Figure 7).

**Figure 8:**
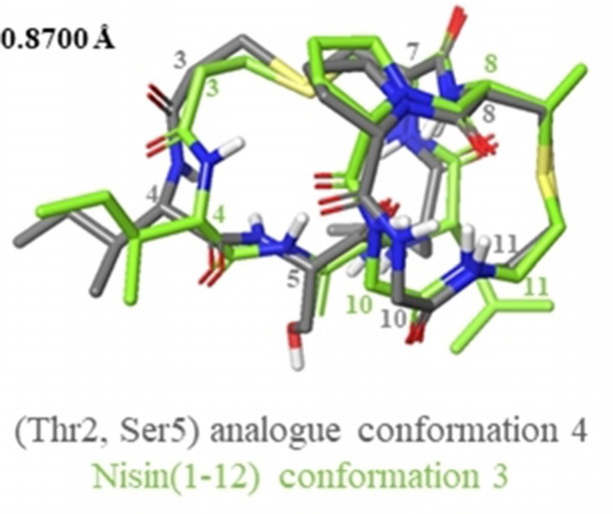
Overlay of (Thr2, Ser5) analogue conformer 4 with nisin(1–12) conformation 3.

Further comparison of the solution ensemble of (Thr2, Ser5) analogue (**2**) to that of nisin(1–12) (**1**) revealed that there is only one similar conformation between the two peptides: (Thr2, Ser5) analogue (**2**) conformation 4, which has similar backbone conformation to the highest populated conformation selected for nisin(1–12) (**1**) (Figure 8). No conformations similar to the WT nisin lipid II‐bound conformation (PDB ID 1WCO)^20^ were selected by NAMFIS analysis of the (Thr2, Ser5) analogue (**2**) ensemble. Indeed, even when the lipid II‐bound conformation from the PDB was included in the starting theoretical ensemble, it was not selected as part of the NAMFIS solution.

This indicates that the (Thr2, Ser5) analogue (**2**) may be less likely to adopt the lipid II‐bound conformation in solution, though the fact that full length nisin Dha5Ser mutants have been shown to maintain MIC values comparable to the native peptide indicates that **2** should still be an effective lipid II binder.^6^ Interestingly, four of the (Thr2, Ser5) analogue (**2**) conformations contain a *cis*Pro (conformations 1, 2, 3, and 5, total 71 %), compared to only one conformation in nisin(1–12) (**1**) (conformation 1, 11 %). Indeed, the short experimental distance between Pro Hα and Lan8 Hα in the (Thr2, Ser5) analogue (**2**) suggested that a *cis*Pro was likely to be present in at least one of the selected conformations.

### Assessing the conformational effect of separating the A and B rings

**Figure 9:**
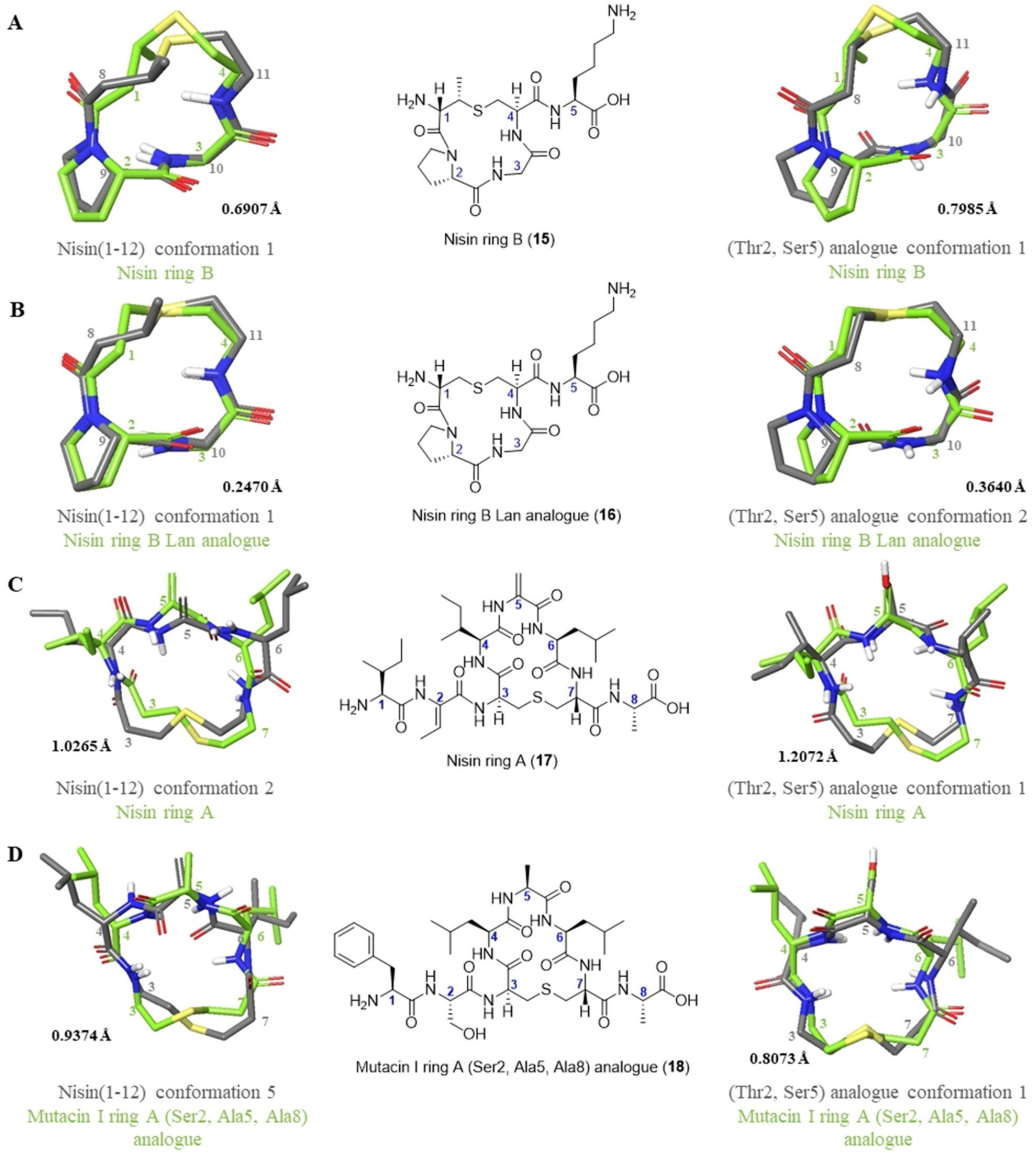
Overlay of averaged solution phase structures^25^ calculated in XPLOR‐NIH^62^, ^63^ (green) with the corresponding regions of nisin(1–12) or (Thr2, Ser5) analogue from NAMFIS analysis (grey).

Previously, we have reported the synthesis and average solution phase conformations (calculated in XPLOR‐NIH^62^, ^63^) of the individual lipid II‐binding rings of nisin and mutacin I.^25^ We therefore sought to compare the NAMFIS solutions to these previously calculated structures to determine whether the presence of a second ring significantly affects conformation. Firstly, isolated nisin ring B (**15**) and a Lan analogue (**16**) were compared to the corresponding regions of the nisin(1–12) (**1**) and (Thr2, Ser5) analogue (**2**) NAMFIS solutions (Figure 9 A and B). As both **15** and **16** were found to bear a *cis*Pro, backbone RMSD was low between these and most of the (Thr2, Ser5) analogue (**2**) conformations, as well as for nisin(1–12) (**1**) conformation 1. Backbone RMSD was not as low when comparing the NAMFIS solutions to nisin ring A (**17**) or mutacin I ring A (Ser2, Ala5, Ala8) analogue (**18**) (Figure 9 C and D). This is presumably due to the increased flexibility made possible by the larger ring size, particularly when Dha5 is replaced for a more flexible residue such as in mutacin I ring A (Ser2, Ala5, Ala8) analogue (**18**). In all cases the largest divergence between the NAMFIS solutions and the isolated rings appears to be in the position of the lanthionine bridge, though the higher flexibility of the lanthionine in nisin ring A is well known in the literature.^14^ Taken together, these results indicate that the structure of each individual lipid II‐binding ring is not particularly affected by the presence of a second ring, though this could be attributed to the relative inflexibility of a small cyclic peptide. However, the results of the NAMFIS analysis presented here indicate that the nisin lipid II‐binding region does exhibit flexibility, though it is predominantly around the central amide bond between the two rings, and that the overall conformation is affected by the nature of the amino acids within each ring.

## Conclusions

Detailed studies of the interactions between the lantibiotic nisin and its biological target, lipid II, require the synthesis of both wild‐type and chemically modified analogues of the key structural moieties. In this paper, we report the first syntheses of two analogues of the bicyclic N‐terminus of nisin, rings A and B, which form a cage‐like structure around the pyrophosphate group of lipid II. Cyclisation of ring A structures from resin‐bound intermediates with ring B in situ proved challenging, probably due to the conformational constraints and partial folding imposed on the key intermediates by the ring B structure. These problems were overcome by the use of pseudoproline residues to induce a turn structure in the linear precursor to ring A, thus facilitating cyclisation and allowing the previously unknown (Thr2, Ser5) nisin(1–12) analogue to be successfully prepared.

However, for bicyclic peptides such as the (Abu2, Ala5) analogue **3**, pseudoproline residues cannot be incorporated into these sequences, and the yield of **3** was thus disappointingly low. The effects of substitution of l‐Ala (and indeed D‐Ala) for Dha at position 5 on the conformation of isolated ring A structures are unclear^25^, ^32^ and the ability to study both **3** and the d‐Abu2, d‐Ala5) analogue would have further confirmed whether such simplified analogues could effectively bind lipid II. This highlights the need for further development of generally applicable methodology for the efficient cyclisation of constrained or polycyclic peptides.

Our motivation for this study was to understand the degree to which the peptide sequence, and the conformational constraints imposed by the two thioether bridges, lead to cage structures that are pre‐organised to bind to the pyrophosphate moiety of lipid II. Previous NMR studies had indicated that individual lanthionine‐bridged ring B structures existed as mixtures of different conformers,^24^, ^64^ however NMR structures of full‐length nisin, either alone or bound to lipid II, show only a single conformer. We have previously shown that isolated ring A structures generally adopt a similar conformation to that observed by NMR for wt nisin in a 1:1 complex with lipid II in solution (1WCO), suggesting a degree of pre‐organisation of ring A.^25^ However, we also demonstrated that isolated ring B structures do not always adopt the conformation observed in the 1WCO NMR structure, although this may be a function of the synthetic methodology used.

In this paper, we have shown that the conformations of each of ring A and ring B are hardly affected by the presence of the other ring, and do not appear to adopt two conformers in the bicyclic structure. However, the nisin‐lipid II binding region exhibits considerable flexibility around the (Lan7, MeLan8) amide bond between the two rings. For the wt nisin(1–12) sequence, this flexibility allows the two rings to fold into the pyrophosphate‐binding cage observed in the 1WCO structure. Conversely, the (Thr2, Ser5) analogue does not appear to form the lipid II‐binding conformation in solution. Perhaps due to additional hydrogen bonding with Ser‐OH, the (Thr2, Ser5) analogue adopts mostly *cis* conformation in solution. These results will inform the rational design of further lipid II‐binding cage structures^35^ which could in turn represent novel lead structures for the development of new antimicrobial peptides.

Our NAMFIS results must also be viewed in the light of recently reported solid state NMR studies^23^ of the 8:4 nisin:lipid II pore in both model liposomes and in membrane vesicles derived from *Micrococcus flavus*. These have suggested that the 1:1 nisin:lipid II structure^20^ may not report on a physiologically relevant state. In particular, there were major perturbations of the chemical shifts of protons in the nisin(1–12) region between the solution state and solid state NMR structures, indicating that rings A and B adopt different conformations in the 1:1 complex solution structure compared with the 8:4 pore structure in the solid state. The observed discrepancies may stem from the different stoichiometries of the complexes, additional constraints imposed by the pore structure, the differences in environment between DMSO and a membrane‐bound complex, or differences between the solution and solid states. In addition, NMR studies on the binding of the structurally unrelated lantibiotic nukacin ISK‐1 to lipid II^65^ have shown that this lantibiotic exists in two conformational states, but with only one conformation capable of binding to lipid II. Intriguingly, the two conformational states also differ in terms of the relative orientation of the two lanthionine‐bridged rings that coordinate to the pyrophosphate moiety. The NAMFIS deconvolution method has previously been used to analyse the ensemble of conformations of epothilones,^66^ and of geldanamycin and radicicol.^67^ In both cases, the presence (or absence) of the receptor‐bound conformation in the NAMFIS ensemble was used to assess the plausibility of conflicting solid‐state and solution structures, and the conformations could then be used as docking candidates for predicting the experimental binding poses in ligand‐receptor complexes. In our work, 26 % of the conformations (conformations 5 and 6) do correspond to the 1WCO 1:1 complex structure, but the most populated set of conformations (conformations 3 and 4, Figure S4: 52 %) do not correspond to the lipid II‐bound conformation observed in the 1WCO structure. Our results suggest that the 1:1 complex solution structure represents one plausible binding mode for the nisin:lipid II interaction. However, there is another favourable set of conformations available to this bicyclic ring structure. These might correspond to an energetically favourable unbound state, as in the nukacin ISK‐1 structure, or to the conformations present in the nisin:lipid II 8:4 solid state pore structure. Our analysis of the conformational states present in the solution ensemble may enable these two possibilities to be distinguished, and will lead to a deeper understanding of the complex factors governing the nisin:lipid II interaction in different environments.

## Experimental Section

Experimental details, including procedures for peptide synthesis, full characterisation data for (Thr2, Ser5) analogue **2** and (Abu2, Ala5) analogue **3**, preparation of wt nisin(1–12), complete assignment of NMR peaks, NAMFIS analysis, NOE build‐up, MCMM conformational search, solution ensemble from NAMFIS algorithm, comparison of all conformations in each NAMFIS solution, comparison of 1WCO backbone structures, are provided in the Supporting Information. In addition, the coordinates for the simulated peptide conformations are also included in the Supporting Information.

## Conflict of interest

The authors declare no conflict of interest.

## Supporting information

As a service to our authors and readers, this journal provides supporting information supplied by the authors. Such materials are peer reviewed and may be re‐organized for online delivery, but are not copy‐edited or typeset. Technical support issues arising from supporting information (other than missing files) should be addressed to the authors.

Supplementary
