# Supplementary material for: A Chemical Biology Approach to Understanding Molecular Recognition of Lipid II by Nisin(1–12): Synthesis and NMR Ensemble Analysis of Nisin(1–12) and Analogues
Source: Chemistry. 2019 Oct 10;25(64):14572–82. doi: 10.1002/chem.201902814 (PMC6899958; doi:10.1002/chem.201902814)
Supplement: Supplementary file 1 — Supplementary [file CHEM-25-14572-s001.pdf]

CHEMISTRY
A European Journal

Supporting Information

A Chemical Biology Approach to Understanding Molecular Recognition of Lipid II by Nisin(1–12): Synthesis and NMR Ensemble Analysis of Nisin(1–12) and Analogues

Rachael Dickman,[a] Emma Danelius,[b] Serena A. Mitchell,[a] D. Flemming Hansen,[d] Máté Erdélyi,*[b, c] and Alethea B. Tabor*[a]

chem_201902814_sm_miscellaneous_information.pdf

Author Contributions

R.D. Investigation: Lead; Writing - Original Draft: Equal; Writing - Review & Editing: Equal
E.D. Investigation: Equal; Writing - Original Draft: Supporting; Writing - Review & Editing: Supporting
S.M. Investigation: Supporting; Writing - Review & Editing: Supporting
D.H. Funding acquisition: Supporting; Methodology: Supporting; Supervision: Supporting; Writing - Review & Editing: Supporting
M.E. Funding acquisition: Equal; Resources: Equal; Supervision: Equal; Writing - Original Draft: Equal; Writing - Review & Editing: Equal
A.T. Conceptualization: Lead; Funding acquisition: Equal; Investigation: Lead; Project administration: Lead; Writing - Original Draft: Lead; Writing - Review & Editing: Lead.

Peptide Synthesis S2

General Information S2

General Methods for Peptide Synthesis S3

Peptide Synthesis S5

WT nisin(1-12) **1** S5

Nisin (Thr2, Ser5) analogue **2** S10

Unsuccessful conversion of **7a** to **8a**, Figure S1 S11

Synthesis of **2** via pseudoproline strategy S12

Nisin (Abu2, Ala5) analogue **3** S16

NAMFIS Analysis S19

NOE Build-Up S19

MCMM Conformational Search S23

Solution Ensemble from NAMFIS Algorithm S23

Figure S6: Comparison of all conformations in each NAMFIS solution S29

Figure S7: Comparison of 1WCO backbone structures S30

References S30

Peptide Synthesis

General Information

The (Teoc, TMSE/Fmoc) lanthionine (**5**) used in peptide syntheses was prepared according to previously described protocols. [1,2]

Peptides were synthesised by hand using the Fmoc solid-phase synthesis strategy. All residues were added to the peptides manually. The resin was continually agitated throughout coupling, deprotection and cleavage steps by shaking at 480 rpm on an IKA KS130 basic platform shaker. Microwave reactions were conducted using a Personal Chemistry Smith Creator microwave-assisted organic synthesizer system in 5 mL reaction vials. Single mode irradiation with monitoring of temperature, pressure and irradiation power versus time was used, with maximum 300 W power. The reaction temperature was kept constant throughout the reaction in the single mode cavity by an automatic power control. An Eppendorf centrifuge model 5810R was used for centrifugation of peptide products before freeze drying by a SP Scientific VirTis BenchTop Pro. All steps not conducted in the microwave oven were performed whilst shaking at room temperature in a 5 mL PP reaction syringe with a frit. The resin was washed copiously with DMF following each coupling and deprotection. Washing the resin refers to the addition of solvent to the resin followed by immediate evacuation.

Peptides were purified by preparative reverse phase HPLC on a Dionex 580 HPLC System with PDA-100 photodiode array detector, P580 Pump and a model ASI-100 automated sample injector. A Phenomenex Onyx C18 100 x 10 mm column or a Dr Maisch GmbH Reprosil Gold 200 C8 5µm 150 x 10 mm column were used (as stated), with detection at 214 and 254 nm. Water (0.1% TFA) and acetonitrile (0.1% TFA) were used as solvents. Chromatograms were analysed using Chromeleon Software version 2.0. Analytical HPLC of peptides was performed on the above described machine or an Agilent Technologies 1260 Infinity system using either an ACE5 C18-300 150 x 4.6 mm column, or a Dr Maisch GmbH Reprosil Gold 200 C8 5µm 250 x 4.6 mm column, with detection at 214 and 254 nm. A linear solvent gradient of 2-98% MeCN (0.1% TFA) in H_2O (0.1% TFA) over 15 min was used, at a flow rate of 1 mL min^{-1}.

LCMS spectra were recorded on a Waters Acquity UPLC SQD using a linear gradient of 5-95% B over 5 min (A = water, B = acetonitrile, 0.1% formic acid) with a C8 column, flow rate 0.6 mL

min^{-1}. Analysis of the chromatograms was conducted using MassLynx software. HRMS spectra were run by King's College Mass Spectrometry Service, Britannia House, on a Waters Xevo G2-XS QTof instrument with ESI source, attached to a Waters Acquity UPLC system (I class) with an Acquity UPLC BEH C18, 1.7 µm column.

All 1D and 2D NMR spectra were recorded on a Bruker Avance-600 spectrometer or Bruker 900 Avance III HD NMR spectrometer equipped with a TCI cryogenic probe, with chemical shifts (δ) given in ppm relative to the solvent residual signal, and coupling constants (*J*) given in Hz. To prepare peptide samples for NMR, the lyophilised powders were dissolved in 0.3 mL anhydrous deuterated DMSO-d_6 which was purchased from Sigma-Aldrich Co. Ltd.. Proton assignments were derived from COSY, TOCSY, NOESY, HSQC and HMBC NMR spectra recorded at 25 °C. $^3J_{HN\text{-}HA}$ coupling constants were measured from 1D ^{1}H NMR spectra. Although not stereospecifically assigned, in WT nisin(1-12), the two Leu δ methyl groups and their corresponding carbon shifts could be distinguished from each other, and the coupling pairs are denoted 'a' and 'b'. Data processing was carried out using ACD/NMR Processor Academic Edition, version 12.01 (Advanced Chemistry Development Inc.) and MNova NMR, version 12.0.3 (Mestrelab Research).

General Methods for Peptide Synthesis

The exact masses and volumes of amino acid, coupling reagent and base used per coupling step are tabulated under the entry for each peptide synthesised.

1. Swelling the resin:
   DMF (2 mL) was added to the resin in a syringe and shaken for 30 min. The DMF was then evacuated and the resin washed with DMF (2 x 2 mL).
2. Fmoc Deprotection:
   A solution of piperidine in DMF (40% v/v, 1.5 mL) was added to the resin and left to shake for 3 min. After this time the syringe was evacuated. Another portion of piperidine in DMF (20% v/v, 1.5 mL) was then added to the resin and left to stir for 10 min. This was evacuated and the resin washed with DMF (6 x 2 mL).
3. Lanthionine coupling steps:

The protected lanthionine **5** (3 eq), HOAt (5 eq) and PyAOP (5 eq) were dissolved in DMF (2 mL) in a glass vial and DIPEA (10 eq) was added. This solution was left to preactivate for 2 min and then, along with the resin, was transferred to a microwave vial and coupled in the microwave at 60 °C for 5 min followed by a further 1 h stirring at rt. The resin and coupling solution were then transferred back to the reaction syringe, the coupling solution was removed and the resin washed thoroughly with DMF (4 x 2 mL).

4. Elongation steps:

   Double coupling of normal Fmoc protected amino acids.

   The desired Fmoc protected amino acid (5 eq), HOAt (5 eq) and PyAOP (5 eq) were dissolved in DMF (1.5 mL for 50 mg scale reactions, 2 mL for 100 and 150 mg scale reactions) and DIPEA (10 eq) was added. This solution was left to preactivate for 2 min and then added to the syringe containing the resin. The suspension was stirred at rt for 2 h before removal of the coupling solution. A fresh sample of the same preactivated coupling solution was then added to the resin and left to stir for 2 h before evacuation. The resin was then washed with DMF (4 x 2 mL).

5. Ring closing steps:

   a. The silyl protecting groups were first removed. A solution of TBAF (1M in THF, 1 mL) in DMF (1 mL) was added to the resin and left to stir at rt under Ar for 1 h. After this time the TBAF solution was removed and the resin washed with DMF (6 x 2 mL).

   b. The terminal Fmoc group was then removed as described above.

   c. A solution of HOAt (5 eq), PyAOP (5 eq) and DIPEA (10 eq) in DMF (1.5 mL for 50 mg scale reactions, 2 mL for 100 and 150 mg scale reactions) was preactivated and then, along with the resin, was transferred to a microwave vial and coupled under microwave irradiation at 60 °C for 5 min followed by a further 1 h stirring at rt. The resin and coupling solution were then transferred back to the reaction syringe, and the coupling solution evacuated before the addition of a fresh solution of activated coupling reagents to the resin and leaving to stir for 2 h. The coupling solution was then removed and the resin washed with DMF (4 x 2 mL).

6. Cleavage:

   Cleavage cocktail:

   TFA (965 µL), water (25 µL), TIPS (10 µL)

The resin was washed with CH_2Cl_2 (3 x 2 mL), MeOH (2 x 2 mL) and ether (2 x 2 mL) and then dried *in vacuo* for 30 min before cleavage. The cleavage cocktail (1 mL) was pre-mixed in a glass vial before addition to the resin. This was left to shake for 40 min before evacuating directly into a 15 mL Falcon tube containing cold ether (7 mL). A further portion of cleavage cocktail (1 mL) was then also added to the resin and left to shake for 30 min before adding to the Falcon tube. The volume was then made up to 14 mL with more cold ether.

This was centrifuged at 4000 rpm at 5 °C for 15 min, after which time a precipitate formed. The ether was poured off and fresh ether added to resuspend the pellet, before a further round of centrifugation (4000 rpm, 5 °C, 10 min). The resuspension and centrifugation process was repeated once more. The resultant pellet was dissolved in water (3 mL) and lyophilised to yield the crude peptide.

Peptide Synthesis

WT Nisin(1-12) (1)

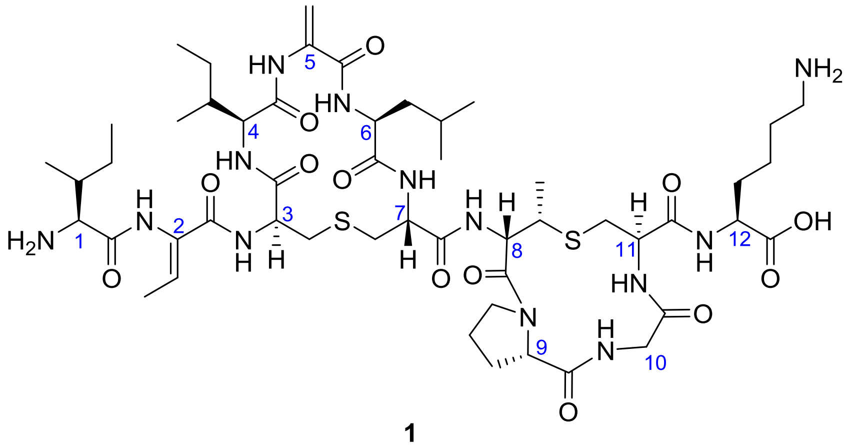

1

Enrichment

A commercially available nisin preparation (20 g) was stirred vigorously in deionised water (500 mL) for 15 min before the addition of CH_2Cl_2 (400 mL). The resulting suspension was transferred to Falcon tubes and centrifuged (2500 rpm, 15 min). The liquid was then decanted and the pellets dried *in vacuo*, before redissolving in deionised water (250 mL) and filtering through Celite®. The solution was then concentrated *in vacuo* to a volume of 50 mL before lyophilisation to give the enriched nisin as a pale brown solid (726 mg).

Trypsin Digestion

A sample of enriched nisin (60 mg) was added to a 50 mL Falcon tube and dissolved in acetate buffer (50 mL; 25 mM NaOAc, 5 mM TRIS base, 5 mM $CaCl_2$, adjusted to pH 7 with acetic acid). This was repeated in 5 additional tubes (total volume of dissolved nisin was 360 mg). The solutions were cooled in an ice-water bath before the addition of trypsin (5 mg) to each, and then were incubated at 30 °C for 24 h. An additional portion of trypsin was then added (5 mg) to each tube and the solutions stirred at 30 °C for another 24 h. The addition-incubation cycle was repeated once more (total incubation time 72 h) before combining the solutions and acidifying to pH 4 with HCl. The solvent was then removed *in vacuo*, with the addition of acetonitrile where necessary to prevent excessive foaming. The residue was then redissolved in water and lyophilised.

The crude peptide was purified by preparative reverse phase HPLC using a semi-prep Dr Maisch GmbH Reprosil Gold 200 C8 5µm 150 x 10 mm column. Initially, a linear solvent gradient of 5-40% MeCN (0.1% TFA) in H_2O (0.1% TFA) over 40 min, at a flow rate of 2 mL min^{-1} was used (retention time 34 min). This was followed by a second purification on a semi-prep Phenomenex Onyx C18 100 x 10 mm column, with a linear solvent gradient of 25-40% MeCN (0.1% TFA) in H_2O (0.1% TFA) over 23 min, at a flow rate of 2 mL min^{-1}. The fractions containing the target peptide (retention time 10 min) were collected and lyophilised to give the sample as a fluffy white powder (2.3 mg). Data is in agreement with the literature.[3,4]

m/z (LCMS, ES+) found $[M+2H]^{2+}$ 576.2.

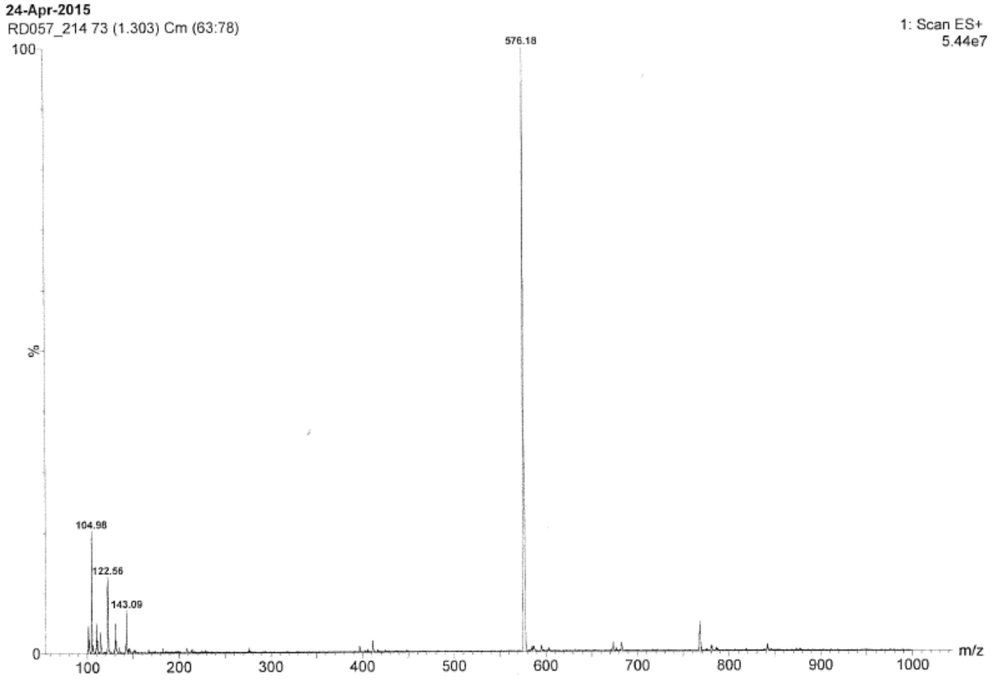

analytical HPLC (ACE5 C8-300 150 x 4.6 mm column on Agilent HPLC system) retention time 8.89 min.

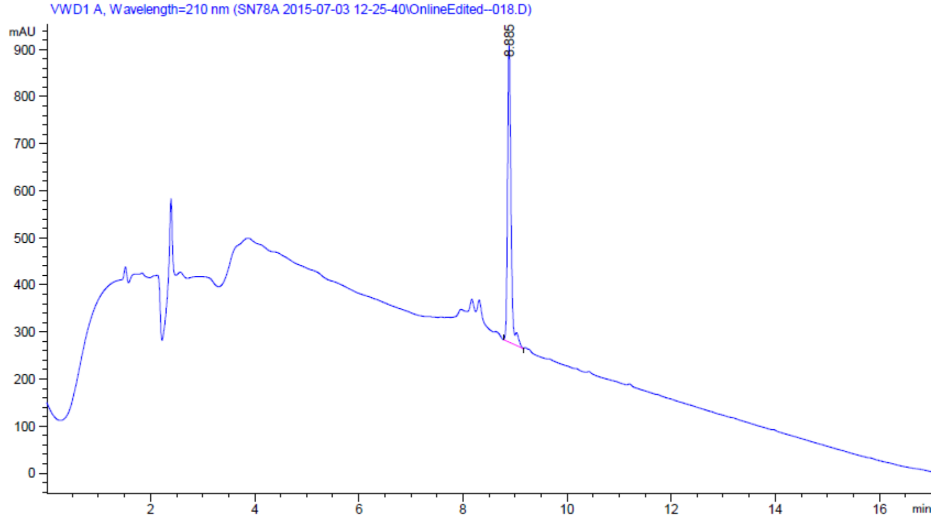

NMR δ_H (900 MHz, $(CD_3)_2SO$), δ_C (150 MHz, $(CD_3)_2SO$)

^1H NMR							
**Residue Number**	**NH**	**α**	**β**	**γ**	**δ**	**ε**	**Exchangeable**
**1 - Ile**		3.79	1.90	CH_2 - 1.19, 1.56<br>CH_3 - 0.98	0.88		NH_2 - 8.13
**2 - Dhb**	9.72		6.28	1.71			
**3 - Lan (Dha)**	8.15	4.39	2.94<br>3.03				
**4 - Ile**	7.91	4.09	2.02	CH_2 - 1.07, 1.38<br>CH_3 - 0.89	0.78		
**5 - Dha**	8.85		5.50<br>5.99				
**6 - Leu**	8.77	4.23	1.55<br>1.64	1.55	a - 0.85<br>b - 0.89		
**7 - Lan (Cys)**	7.95	4.64	2.79<br>3.08				
**8 - MeLan (Dhb)**	8.16	4.17	3.45	1.18			
**9 - Pro**		4.19	1.65<br>2.25	1.89<br>1.76	3.27<br>3.38		
**10 - Gly**	8.25	4.97					
**11 - MeLan (Cys)**	7.59	3.84	2.97<br>3.39				
**12 - Lys**	8.21	4.18	1.75	1.34	1.55	2.76	NH_2 - 7.64<br>CO_2H - 12.70

^{13}C NMR						
**Residue Number**	**CO**	**α**	**β**	**γ**	**δ**	**ε**
**1 - Ile**	175.92	59.60	39.19	CH_2 - 27.33<br>CH_3 - 17.84	14.26	
**2 - Dhb**	167.39	133.13	129.70	16.23		
**3 - Lan (Dha)**	175.42	57.01	37.87			
**4 - Ile**	172.83	61.02	36.62	CH_2 - 27.54<br>CH_3 - 18.87	13.80	
**5 - Dha**	167.83	137.79	107.23			
**6 - Leu**	175.69	54.57	33.61	27.62	a - 24.80<br>b - 25.96	
**7 - Lan (Cys)**	173.58	57.39	42.77			
**8 - MeLan (Dhb)**	176.28	60.24	51.43	25.01		
**9 - Pro**	173.94	66.08	31.81	29.38	50.90	
**10 - Gly**	174.43	59.89				
**11 - MeLan (Cys)**	172.25	56.63	39.45			
**12 - Lys**	176.87	54.52	41.05	25.54	29.55	41.76

**Residue Number**	$^{3}J_{HA-HN}$
**4 - Ile4**	7.9
**6 - Leu**	7.8
**7 - Lan**	7.5
**12 - Lys**	8.1

Nisin (Thr2, Ser5) Analogue (2)

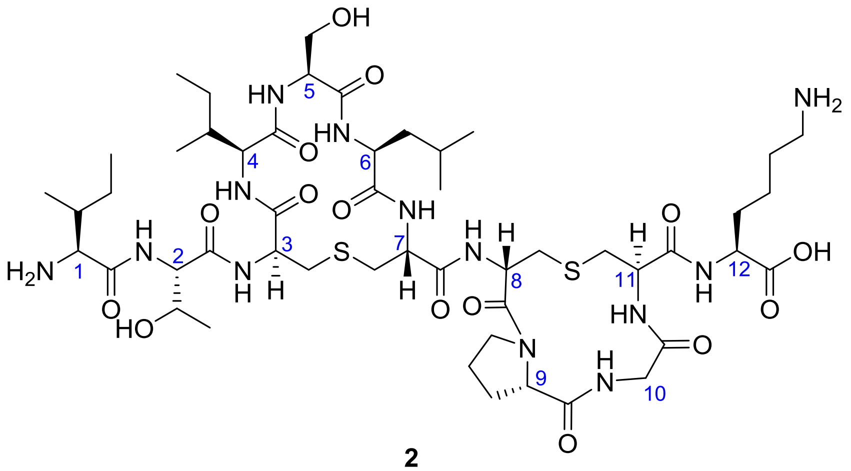

2

Reagents		Mass/Volume	µmol
**Amino Acids**	Teoc/TMSE lanthionine	37 mg	54
	Fmoc-Gly-OH	27 mg	90
	Fmoc-Pro-OH	30 mg	90
	Fmoc-Leu-OH	32 mg	90
	Fmoc-Ile-Ser($\psi^{Me,Me}$pro)-OH	43 mg	90
	Fmoc-Thr(*t*Bu)-OH	36 mg	90
	Fmoc-Ile-OH	32 mg	90
**Coupling Reagents**	HOAt	12 mg	90
	PyAOP	47 mg	90
	DIPEA	31 µL	180

Unsuccessful conversion of 7a to 8a

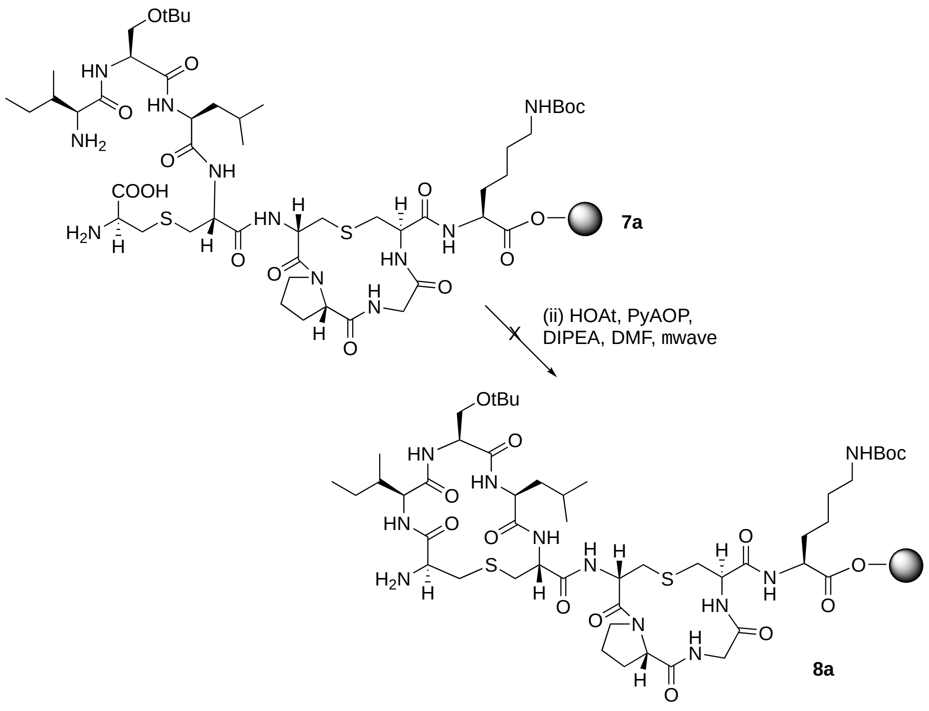

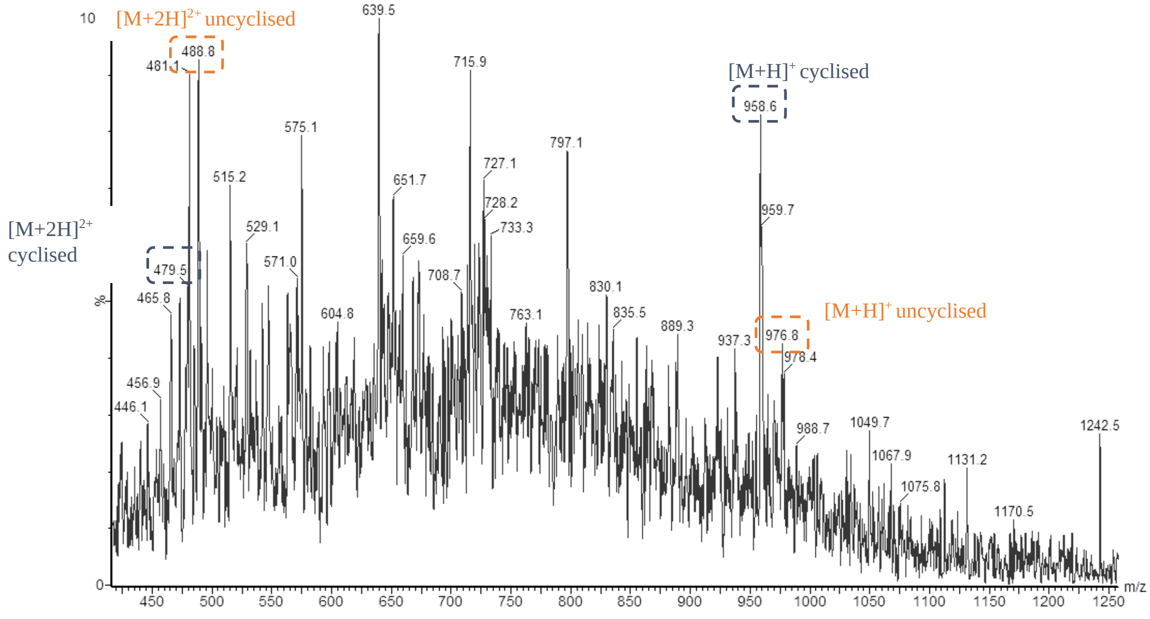

Figure S1: LCMS trace of mini cleave experiment taken after attempted cyclisation of **7a** to **8a**. Mini cleave conditions: a small portion of resin (~ 1 mg) was removed and treated with neat TFA (200 uL). After 20 min, the solution was diluted with water and analysed by LCMS.

*SPPS of (Thr2, Ser5) analogue **2** using a pseudoproline strategy*

Fmoc-Lys(Boc)-NovaSyn TGT resin (100 mg, 18.0 μmol) was swollen in DMF and the Fmoc group removed as described in General Methods Sections 1 and 2.

The protected Teoc/TMSE lanthionine was coupled using the microwave procedure described in General Methods Section 3. The Fmoc group was then removed.

The peptide chain was elongated using the double coupling procedure described in General Methods Section 4. Fmoc-Gly-OH was added first, the Fmoc group was removed, followed by the addition of Fmoc-Pro-OH.

The cyclisation reaction was then carried out as described in Section 5. The silyl protecting groups were removed (General Methods Section 5a), followed by removal of the Fmoc group (General Methods Section 5b). The cyclisation reaction was then carried out as described in Section 5c for lanthionine containing peptides.

A second Teoc/TMSE lanthionine was then coupled onto the B ring using the microwave procedure described in General Methods Section 3. The Fmoc group was then removed and the peptide chain further elongated using the double coupling procedure described in General Methods Section 4. Fmoc-Leu-OH was added first and the Fmoc group was removed, followed by the addition of Fmoc-Ile-Ser($\psi^{Me,Me}$pro)-OH. The second cyclisation reaction was then carried out in the same way as the first, as described in General Methods Section 5.

The peptide chain was then further elongated using the double coupling procedure described in General Methods Section 4. Firstly Fmoc-Thr(*t*Bu)-OH was added and the Fmoc group removed, followed by the addition of Fmoc-Ile-OH, and removal of the final Fmoc group.

Following completion of the synthesis, the peptide was cleaved from the resin using the procedure described in General Methods Section 6.

The crude peptide (9.5 mg) was then transferred to a glass vial and cooled in ice. Triflic acid (200 μL) was added dropwise and the solution agitated until the peptide was dissolved. The solution

was left in ice for 5 min before transferring the whole reaction mixture to a Falcon tube containing cold ether (10 mL) and centrifuging at 4000 rpm at 5 °C for 15 min, after which time a precipitate formed. The ether was poured off and fresh ether added to resuspend the pellet, before a further round of centrifugation (4000 rpm, 5 °C, 10 min). The resuspension/centrifugation process was repeated once more. The resultant pellet was resuspended in water (3 mL) and lyophilised.

The crude peptide was purified by preparative reverse phase HPLC using a semi-prep Phenomenex Onyx C18 100 x 10 mm column with a linear solvent gradient of 20-60% MeCN (0.1% TFA) in H_2O (0.1% TFA) over 28 min. A second round of purification was then carried out on the Agilent HPLC system using a Dr Maisch GmbH Reprosil Gold 200 C8 5µm 250 x 4.6 mm column and a gradient of 15-25% MeCN (0.1% TFA) in H_2O (0.1% TFA) over 35 min. The fractions containing the target peptide (retention time 23-24 min) were collected and lyophilised to give the pure sample as a white powder (600 µg, 3%).

analytical HPLC (Dr Maisch GmbH Reprosil Gold 200 C8 5µm 250 x 4.6 mm column on Agilent HPLC system) retention time 7.69 min.

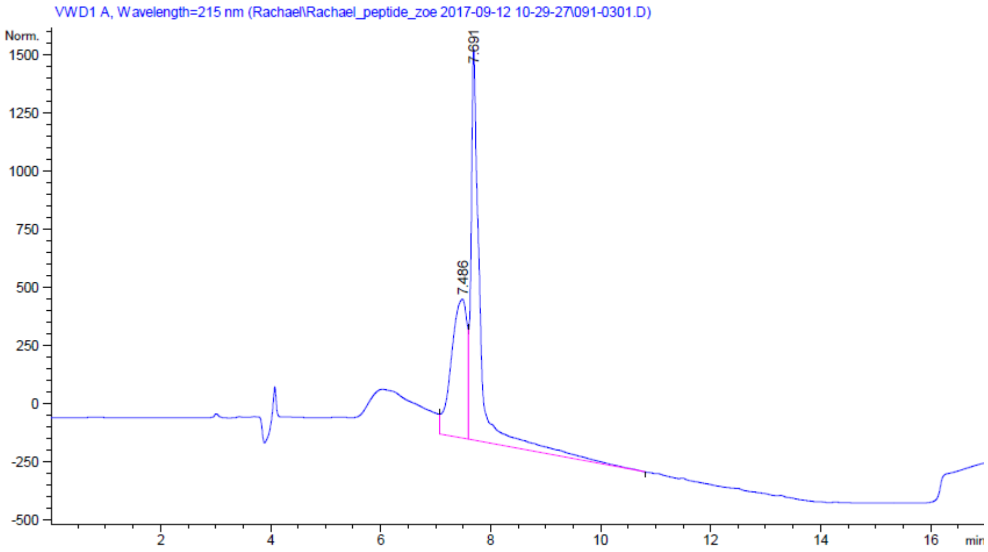

m/z (HRMS, ES+) required for $[C_{50}H_{86}N_{13}O_{15}S_2]^+$ 1172.5808, found $[C_{50}H_{86}N_{13}O_{15}S_2]^+$ 1172.5796.

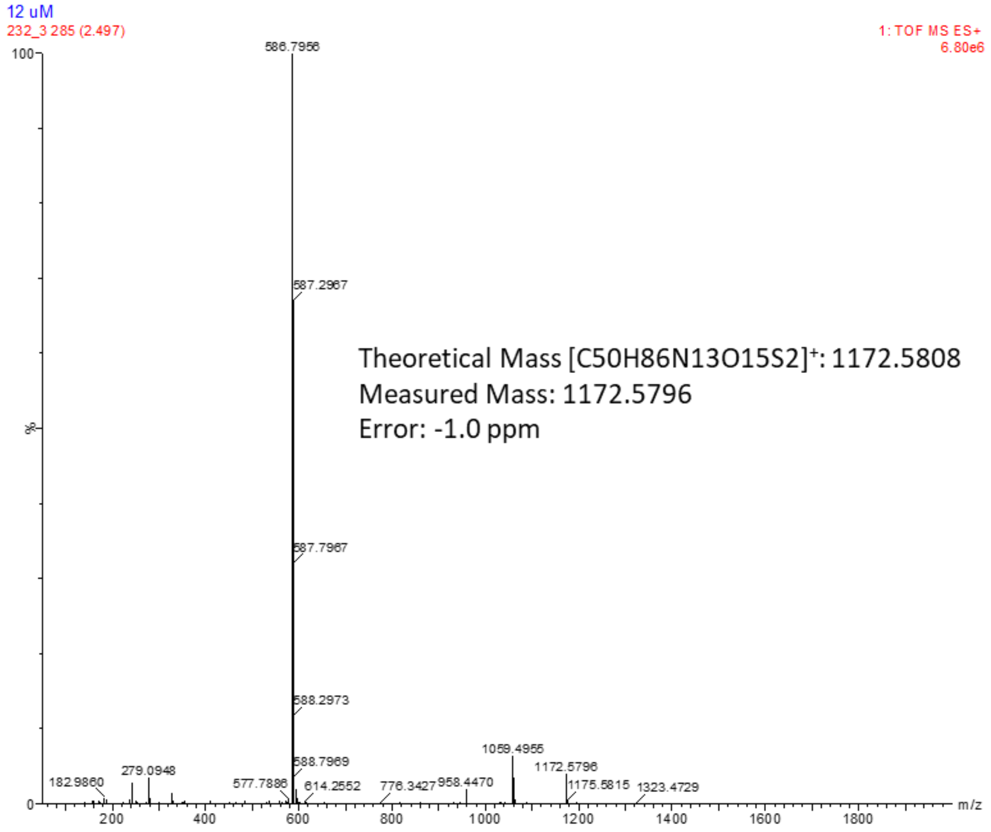

NMR δ_H (900 MHz, $(CD_3)_2SO$)

Some shifts of ring A residues were not observed, these are indicated by 'n.o'

^{1}H NMR						
**Residue Number**	**NH**	α	β	γ	δ	ε
**1 - Ile**	-	n.o	1.77	CH_2 - 1.13, 1.52, CH_3 - 0.90	0.85	
**2 - Thr**		3.97	4.45	1.16		
**3 - Lan (Dha)**	8.43	4.41	3.22<br>2.90			
**4 - Ile**	7.72	3.92	1.79	CH_2 - 0.99, 1.31, CH_3 - 0.81	0.74	
**5 - Ser**	7.90	4.20	3.58<br>3.68			
**6 - Leu**	n.o	n.o	n.o	1.52	0.84<br>0.90	
**7 - Lan (Cys)**	n.o	n.o	n.o			
**8 - Lan (Dha)**	8.53	4.58	2.55<br>2.86			
**9 - Pro**	-	4.84	2.26<br>1.95	1.8, 1.77	3.48<br>3.37	
**10 - Gly**	8.72	3.63<br>3.72				
**11 - Lan (Cys)**	7.55	4.57	2.94<br>2.57			
**12 - Lys**	8.30	4.12	1.72	1.34	1.53<br>1.58	2.76

**Residue Number**	$^3J_{HA\text{-}HN}$
**4 - Ile4**	7.6
**12 - Lys**	9.2

Nisin (Abu2, Ala5) Analogue (3)

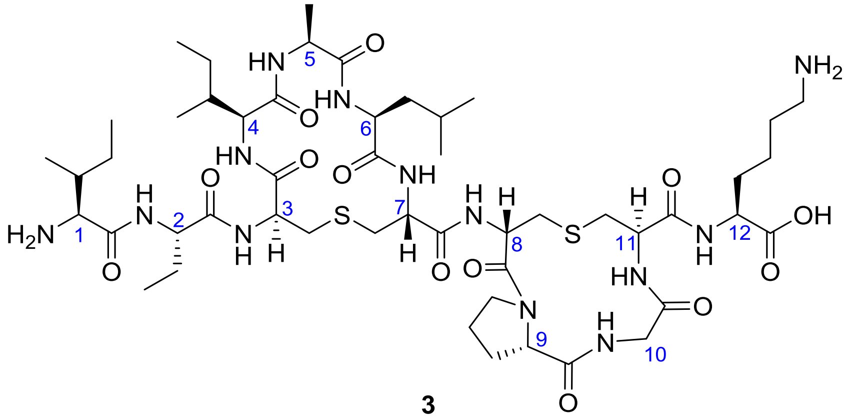

3

Reagents		Mass/Volume	μmol
**Amino Acids**	Teoc/TMSE lanthionine	37 mg	54
	Fmoc-Gly-OH	27 mg	90
	Fmoc-Pro-OH	30 mg	90
	Fmoc-Leu-OH	32 mg	90
	Fmoc-Ala-OH	28 mg	90
	Fmoc-Ile-OH	32 mg	90
	Fmoc-Abu -OH	29 mg	90
**Coupling Reagents**	HOAt	12 mg	90
	PyAOP	47 mg	90
	DIPEA	31 μL	180

Fmoc-Lys(Boc)-NovaSyn TGT resin (100 mg, 18.0 μmol) was swollen in DMF and the Fmoc group removed as described in General Methods Sections 1 and 2.

The protected Teoc/TMSE lanthionine was coupled using the microwave procedure described in General Methods Section 3. The Fmoc group was then removed.

The peptide chain was elongated using the double coupling procedure described in General Methods Section 4. Fmoc-Gly-OH was added first, the Fmoc group was removed, followed by the addition of Fmoc-Pro-OH.

The cyclisation reaction was then carried out as described in Section 5. The silyl protecting groups were removed (General Methods Section 5a), followed by removal of the Fmoc group (General Methods Section 5b). The cyclisation reaction was then carried out as described in Section 5c for lanthionine containing peptides.

A second Teoc/TMSE lanthionine was then coupled onto the B ring using the microwave procedure described in General Methods Section 3. The Fmoc group was then removed and the peptide chain further elongated using the double coupling procedure described in General Methods Section 4. Fmoc-Leu-OH was added first, the Fmoc group was removed, followed by the addition of Fmoc-Ala-OH, removal of the Fmoc group and coupling of Fmoc-Ile-OH. The second cyclisation reaction was then carried out in the same way as the first, as described in General Methods Section 5.

The peptide chain was then further elongated using the double coupling procedure described in General Methods Section 4. Firstly Fmoc-Abu-OH was added and the Fmoc group removed, followed by the addition of Fmoc-Ile-OH, and removal of the final Fmoc group.

Following completion of the synthesis, the peptide was cleaved from the resin using the procedure described in General Methods Section 6.

The crude peptide was partially purified (76% by HPLC) by preparative reverse phase HPLC using a semi-prep Phenomenex Onyx C18 100 x 10 mm column with a linear solvent gradient of 5-40% MeCN (0.1% TFA) in H_2O (0.1% TFA) over 27 min. A second round of purification was then carried out on the Agilent HPLC system using a Dr Maisch GmbH Reprosil Gold 200 C8 5µm 250 x 4.6 mm column and a gradient of 15-35% MeCN (0.1% TFA) in H_2O (0.1% TFA) over 35 min. The fractions containing the target peptide were collected (retention time 29 min) and lyophilised to give the sample as a white powder (100 µg, 0.5%).

m/z (HRMS, ES+) required for $[C_{50}H_{86}N_{13}O_{13}S_2]^+$ 1140.5909, found $[C_{50}H_{86}N_{13}O_{13}S_2]^+$ 1140.5895.

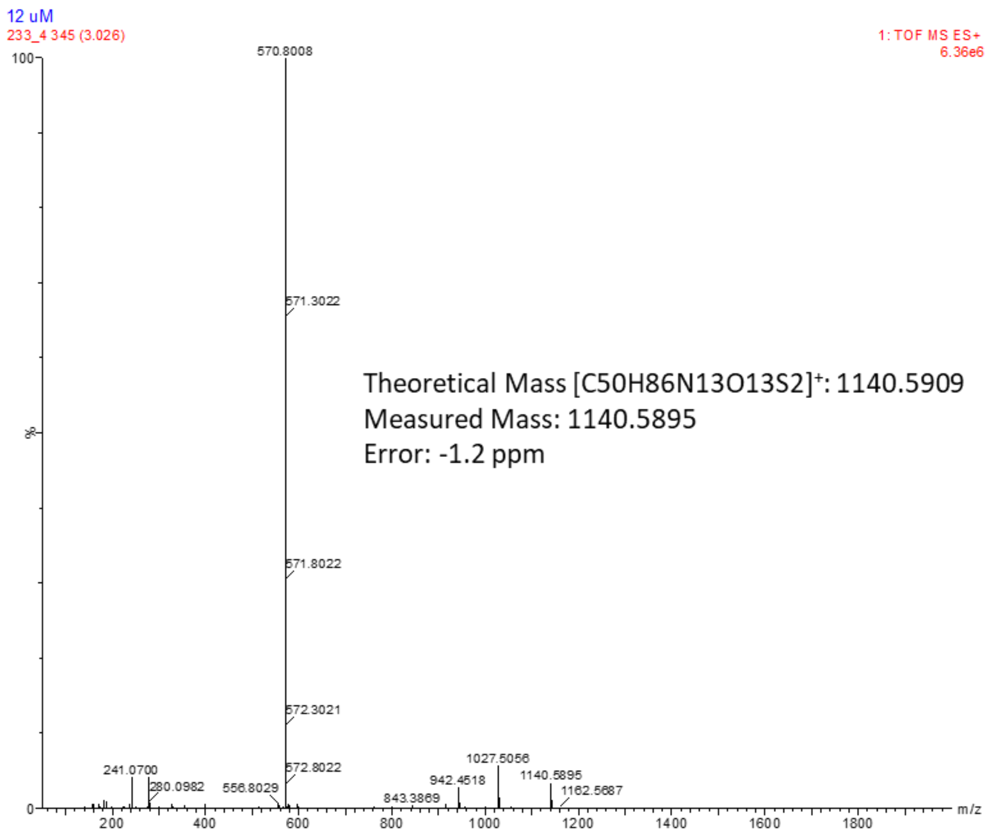

analytical HPLC (Dr Maisch GmbH Reprosil Gold 200 C8 5µm 250 x 4.6 mm column on Agilent HPLC system) retention time 8.35 min.

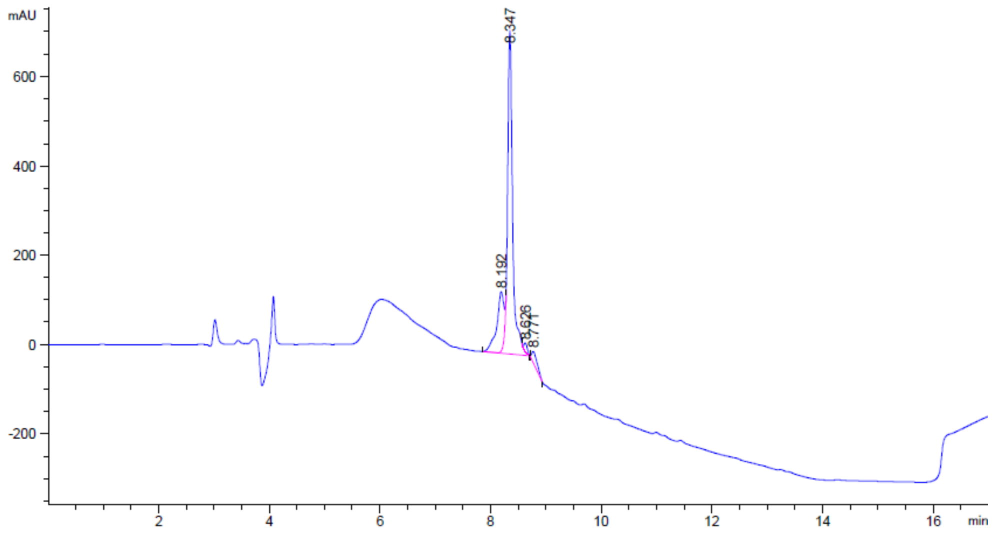

NAMFIS Analysis

NOE Build-Up Analysis

NOE build-ups were recorded without solvent suppression with mixing times of 100, 200, 300, 400, 500, 600 and 700 ms. The relaxation delay was set to 2.5 s, and 16 scans were recorded with 8192 points in the direct dimension and 512 points (nisin(1-12) **1**) or 1024 points ((Thr2, Ser5) analogue **2**) in the indirect dimension. Distances were calculated using geminal methylene protons (1.78 Å) as reference. Comparable distances obtained from different methylene proton pairs served as a measure of data quality. The NOE peak intensities were calculated using normalization of both cross peaks and diagonal peaks according to ([cross peak1 × cross peak2]/[diagonal peak1 × diagonal peak2])$^{0.5}$. At least 6 mixing times giving a linear (r2 > 0.97 for nisin(1-12) (**1**) and r2 > 0.96 for (Thr2, Ser5) analogue **2**) initial NOE rate for every distance were used to determine σ_{ij} build-up rates according to the equation $r_{ij}=r_{ref}(\sigma_{ref}/\sigma_{ij})^{(1/6)}$, where r_{ij} is the distance between protons i and j in Ångström and σ_{ij} and σ_{ref} are the build-up rates based on the normalized intensity obtained from NOESY experiments, for the i and j protons and the reference protons.

Table S1: Interproton distances (Å) for nisin(1-12) **1** derived from NOE build-up measurements in DMSO-d_6.

No.	Proton A	Proton B	δA (ppm)	δB (ppm)	σ	R2	Distance rAB (Å)
**1**	Dha NH	Ile4 NH	8.85	7.92	0.0001921	1.00	2.31
**2**	Leu NH	Lan7 NH	8.77	7.95	0.0000687	0.99	2.74
**3**	Gly NH	Lan7 NH	8.25	7.95	0.0000617	0.99	2.79
**4**	Lys NH	MeLan11 α	8.21	3.84	0.0004979	1.00	1.97
**5**	Lan7 NH	Leu α	7.95	4.23	0.0002931	1.00	2.15
**6**	Ile4 NH	Lan3 α	7.92	4.39	0.0001053	0.99	2.55
**7**	MeLan11 NH	MeLan8 β	7.59	3.45	0.0001325	0.98	2.46
**8**	Dha NH	Lan3 β	8.85	3.03	0.0000123	1.00	3.65
**9**	Gly NH	Pro δ	8.25	3.27	0.0000543	0.99	2.85
**10**	Lan7 NH	Lan7 β	7.95	2.79	0.0001204	0.99	2.50
**11**	Ile4 NH	Lan3 β	7.92	2.93	0.0001217	0.99	2.49
**12**	Lys NH	MeLan11 β	8.21	2.97	0.0000538	0.98	2.86
**13**	MeLan11 NH	MeLan11 β	7.59	2.97	0.0002282	1.00	2.25
**14**	MeLan8 β	MeLan11 β	3.45	2.97	0.0004491	0.98	2.01
**15**	Lan3 α	Lan7 β	4.39	2.79	0.0000873	0.98	2.64

**16**	Leu NH	Dha β	8.77	6.00	0.0002612	0.99	2.20
**Ref.**	Lan3 β	Lan3 β	3.03	2.93	0.0009211	0.97	1.78
**Ref.**	Ile4 $CH_2γ$	Ile4 $CH_2γ$	1.38	1.07	0.0007555	0.99	1.84

Figure S2: Build-up curves for interproton distances of nisin(1-12).

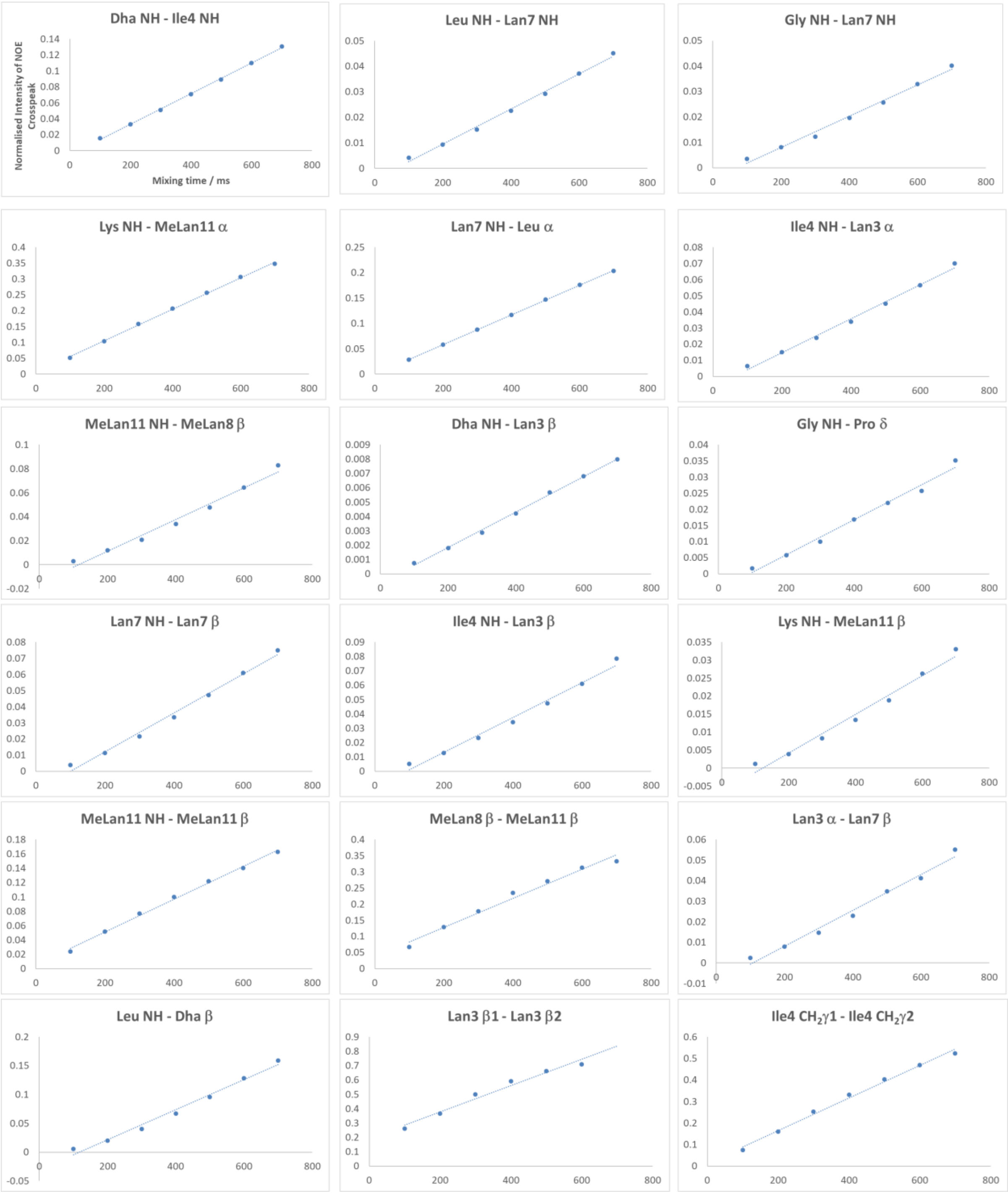

Table S2: Interproton distances (Å) for (Thr2, Ser5) analogue **2** derived from NOE build-up measurements in DMSO-d_6.

**No.**	**Proton A**	**Proton B**	**δA (ppm)**	**δB (ppm)**	**σ**	**R2**	**Distance rAB (Å)**
**1**	Gly NH	Lan11 α	8.72	4.57	0.0000051	0.96	3.77
**2**	Gly NH	Lan11 NH	8.72	7.55	0.0000938	0.99	2.32
**3**	Gly NH	Pro α	8.72	4.84	0.0002677	1.00	1.95
**4**	Lys NH	Lan11 α	8.30	4.57	0.0002597	1.00	1.96
**5**	Lan11 NH	Pro α	7.55	4.84	0.0000415	0.97	2.66
**6**	Pro α	Lan8 α	4.84	4.58	0.0003634	1.00	1.85
**7**	Lan8 NH	Lan8 α	8.53	4.58	0.0000963	0.99	2.31
**8**	Lan 11 NH	Lan11 α	7.55	4.57	0.0002021	0.99	2.04
**9**	Lys NH	Lan11 β	8.30	2.57	0.0000639	0.99	2.48
**10**	Lan11 NH	Gly α	7.55	3.72	0.0000187	0.99	3.04
**11**	Lan11 NH	Lan11 β	7.55	2.57	0.0000636	0.99	2.48
**12**	Gly NH	Gly α	8.72	3.63	0.0001776	0.98	2.09
**13**	Lan8 α	Lan8 β	4.58	2.86	0.0001354	1.00	2.19
**14**	Lan11 α	Lan11 β	4.57	2.94	0.0002456	0.99	1.98
**15**	Lan3 α	Lan3 β	4.41	2.90	0.0001884	1.00	2.07
**Ref.**	Gly α	Gly α	3.63	3.72	0.0004635	0.96	1.78
**Ref.**	Ser β	Ser β	3.58	3.68	0.0003580	0.98	1.86
**Ref.**	Pro β	Pro β	1.95	2.26	0.0006017	0.96	1.70
**Ref.**	Ile4 $CH_2\gamma$	Ile4 $CH_2\gamma$	0.99	1.31	0.0002965	0.99	1.92

Figure S3: Build-up curves for interproton distances of (Thr2, Ser5) analogue **2**.

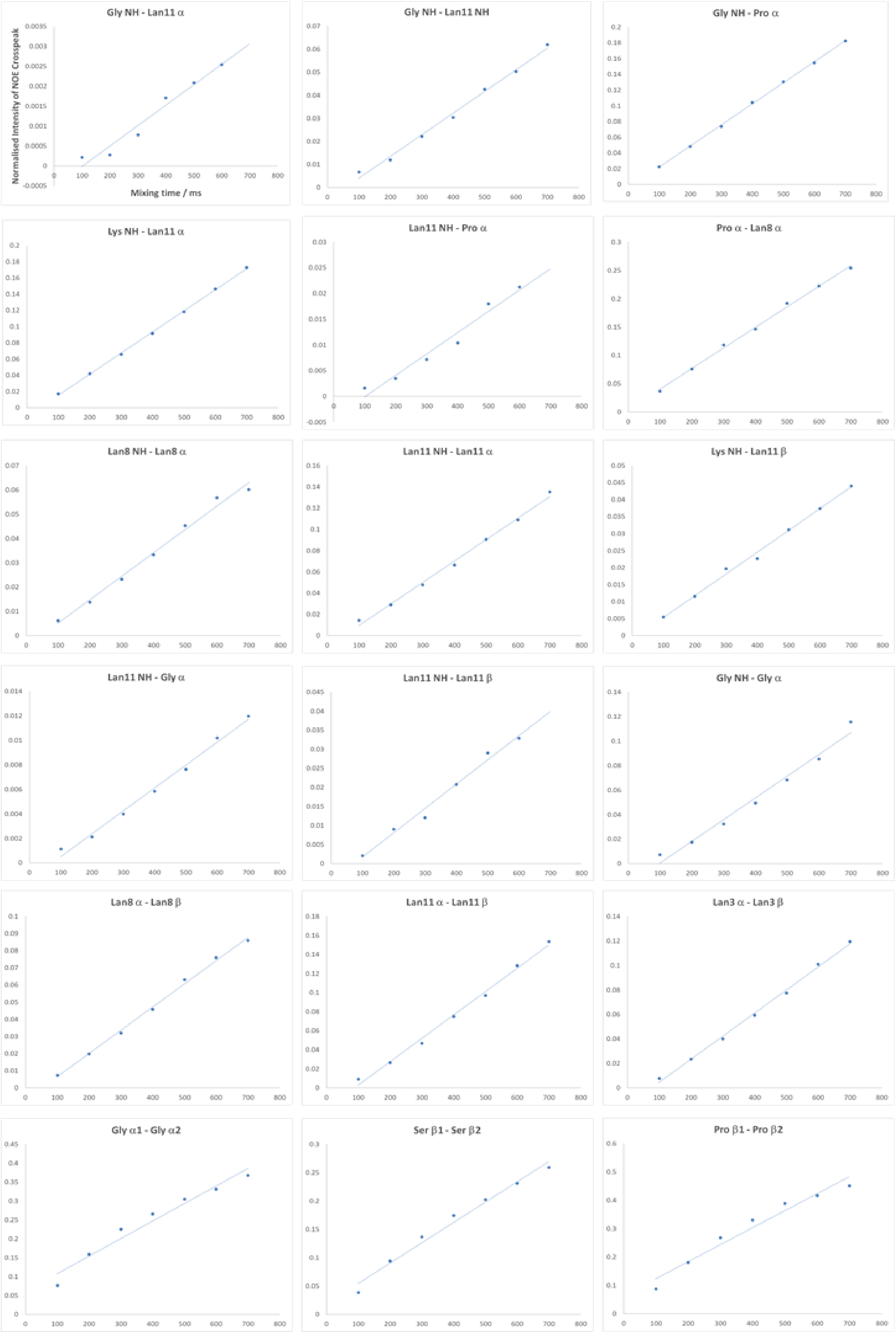
Gly NH - Lan11 α
Normalised Intensity of NOE Crosspeak
Mixing time / ms
Gly NH - Lan11 NH
Gly NH - Pro α
Lys NH - Lan11 α
Lan11 NH - Pro α
Pro α - Lan8 α
Lan8 NH - Lan8 α
Lan11 NH - Lan11 α
Lys NH - Lan11 β
Lan11 NH - Gly α
Lan11 NH - Lan11 β
Gly NH - Gly α
Lan8 α - Lan8 β
Lan11 α - Lan11 β
Lan3 α - Lan3 β
Gly α1 - Gly α2
Ser β1 - Ser β2
Pro β1 - Pro β2

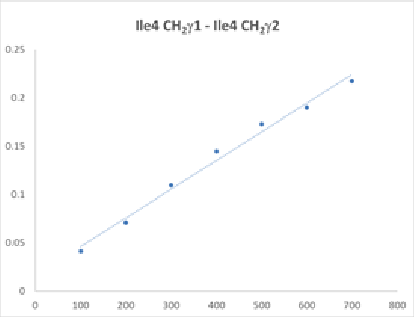

MCMM Conformational Search

In order to provide ensembles covering the available conformational space of the peptides, careful Monte Carlo conformational searches were performed using the OPLS-2005 and the Amber* force fields using an implicit H_2O solvent model (PCM). These conformational searches were performed using the Monte Carlo algorithm with intermediate torsion sampling, 50000 Monte Carlo steps, and a RMSD cut-off set to 2.0 Å, followed by Molecular Mechanics energy minimization, with the software Macromodel (v.9.1) as implemented in the Schrödinger package. The energy minimization was performed using the Polak-Ribiere type conjugate gradient (PRCG) with maximum iteration steps set to 5000. All conformations within 42 kJ/mol from the global minimum were saved. The results of the four independent searches performed using OPLS-2005 or Amber* as force field are given below. To estimate the probability that a conformational search is complete the equation $1-(1-(1/N))^M$ can be used, where N is the total number of conformers and M is the number of search steps.[5] The ensembles generated here fulfilled the equation. Moreover, the seven 'lowest energy' conformations were found on average at least 7 times each, which is also an indicator of search completeness. The ensembles from the conformational searches were combined and elimination of redundant conformations by comparisons of the heavy atom coordinates applying the RMSD cutoff 2.5 Å was performed, giving the input ensemble used for NAMFIS. In the case of the nisin(1-12) ensemble, the lipid II-bound conformation available in the PDB (PDB ID 1WCO) was also added to the ensemble.[6]

Solution Ensemble from the NAMFIS Algorithm

Solution ensembles were determined by fitting the experimentally measured distances and coupling constants to those back-calculated for computationally predicted conformations following previously described protocols.[7] CH_2-signals were treated according to the equation

$d=(((d_1^{-6})+(d_2^{-6}))/2)^{-1/6}$. $^3J_{HN-HA}$ coupling constants were calculated from dihedral angles according to the Karplus equation $J = 9.4 \cos^2 \theta - 1.1 \cos \theta + 0.4$.[8]

Table S3. Results of the MCMM conformational analysis for nisin(1-12) **1** and (Thr2, Ser5) analogue **2**.

			**Number of conformations**	
		Total[a]	Within 12.6 kJ/mol[b]	Following redundant conformer elimination[c]
Nisin(1-12) **1**	OPLS	246	25	125
	Amber*	185	7	
(Thr2, Ser5) analogue **2**	OPLS	206	19	119
	Amber*	90	14	

[a] Total number of unique conformations found. [b] Conformations found within 12.6 kJ/mol (3.0 kcal/mol) of the global minimum. [c] Conformations obtained after redundant conformation elimination with the root-mean-square deviation cutoff 2.5 Å for heavy atoms.

The NAMFIS ensemble analyses were validated using standard methods, that is, through evaluation of the reliability of the conformational restraints by the addition of 10% random noise to the experimental data, by the random removal of individual restraints, and by comparison of the experimentally observed and back-calculated distances. The solution was validated to within ±10% (nisin(1-12) **1**) or ±15% ((Thr2, Ser5) analogue **2**). Figures were generated using the software Maestro (Version 11.4, Schrödinger, LLC).

Table S4. Result of the NAMFIS analyses in DMSO-d_6.

**Nisin(1-12) 1**		**(Thr2, Ser5) analogue 2**	
**Conformation Number [a]**	**% [b]**	**Conformation Number [a]**	**% [b]**
1	11	1	13
2	11	2	7
3	15	3	35
4	37	4	29
5	7	5	14
6	19		

[a]The structures of the most populated conformations are shown in Figure 2. [b]Population of the indicated conformer in solution, all other molar fractions are 2% or less.

Table S5. Experimentally determined and back-calculated (NAMFIS) interproton distances (Å) and coupling constants for nisin(1-12) **1**.

**Interproton Distances**		**Coupling Constants**	
**Experimental**	**Calculated**	**Experimental**	**Calculated**
2.31	2.12	7.9	8.0
2.74	2.96	7.8	7.6
2.79	4.01	7.5	7.4
1.97	2.48	8.1	8.1
2.15	2.73		
2.55	2.80		
2.46	3.08		
3.65	3.27		
2.85	3.19		
2.50	2.99		
2.49	3.18		
2.86	3.29		
2.25	2.88		
2.01	2.74		
2.64	4.42		
2.20	2.17		

Figure S4: The solution conformations of nisin(1-12) **1** in DMSO-d_6 as selected by the NAMFIS-analysis of the MCMM ensemble and lipid II-bound conformation taken from the PDB (PDB ID 1WCO).[6] Population % are given in table S4. Non-polar hydrogens are omitted for clarity.

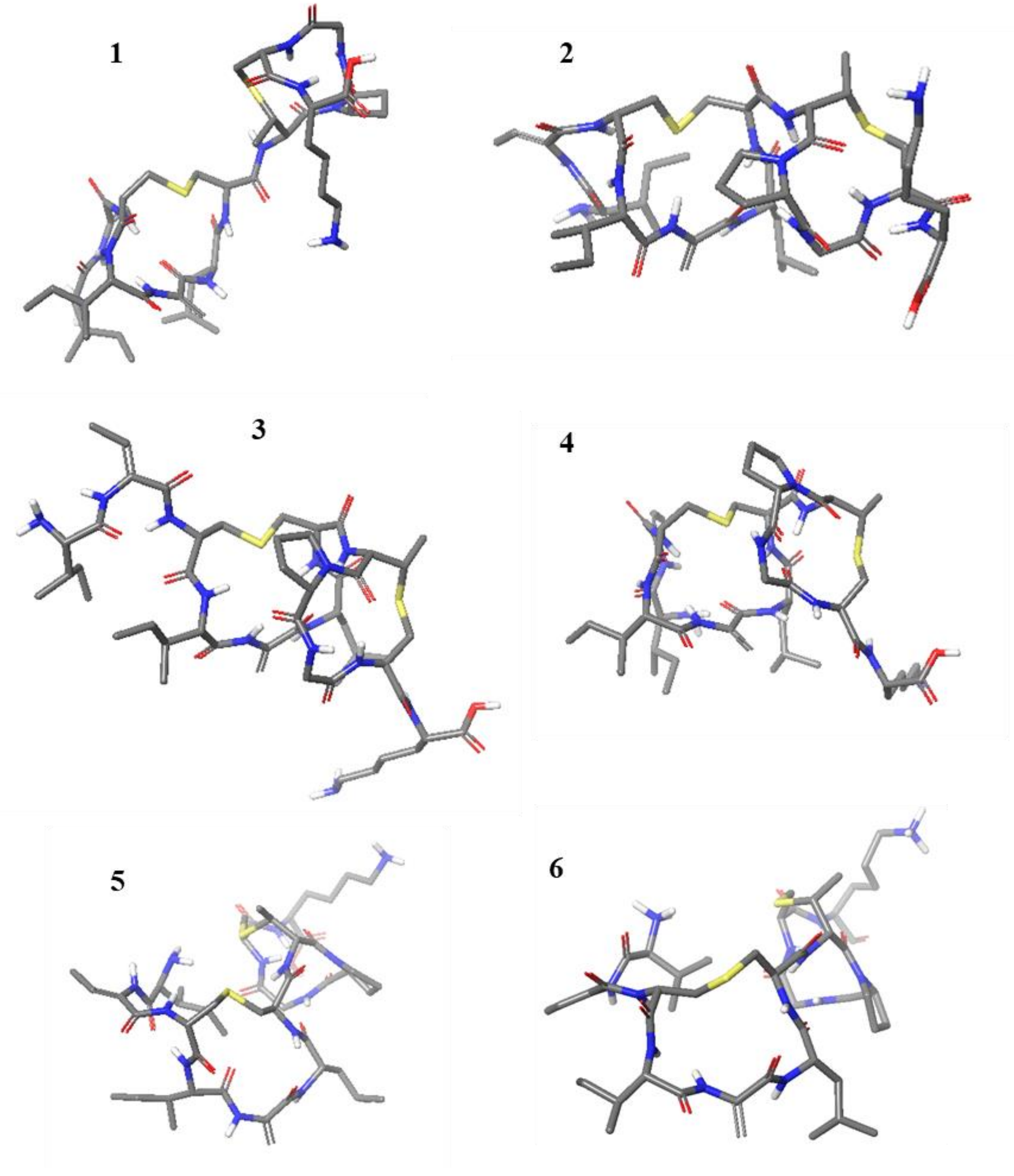

Table S5. Experimentally determined and back-calculated (NAMFIS) interproton distances (Å) and coupling constants for (Thr2, Ser5) analogue **2**.

**Interproton Distances**		**Coupling Constants**	
**Experimental**	**Calculated**	**Experimental**	**Calculated**
3.77	4.95	7.6	7.6
2.32	2.31	9.2	9.2
1.95	2.20		
1.96	2.38		
2.66	3.51		
1.85	2.40		
2.31	2.42		
2.04	2.82		
2.48	2.62		
3.04	3.19		
2.48	2.87		
2.09	2.53		
2.19	2.59		
1.98	2.52		
2.07	2.48		

Figure S5: The solution conformations of (Thr2, Ser5) analogue **2** in DMSO-d_6 as selected by the NAMFIS-analysis of the MCMM ensemble. Population % are given in table S4. Non-polar hydrogens are omitted for clarity.

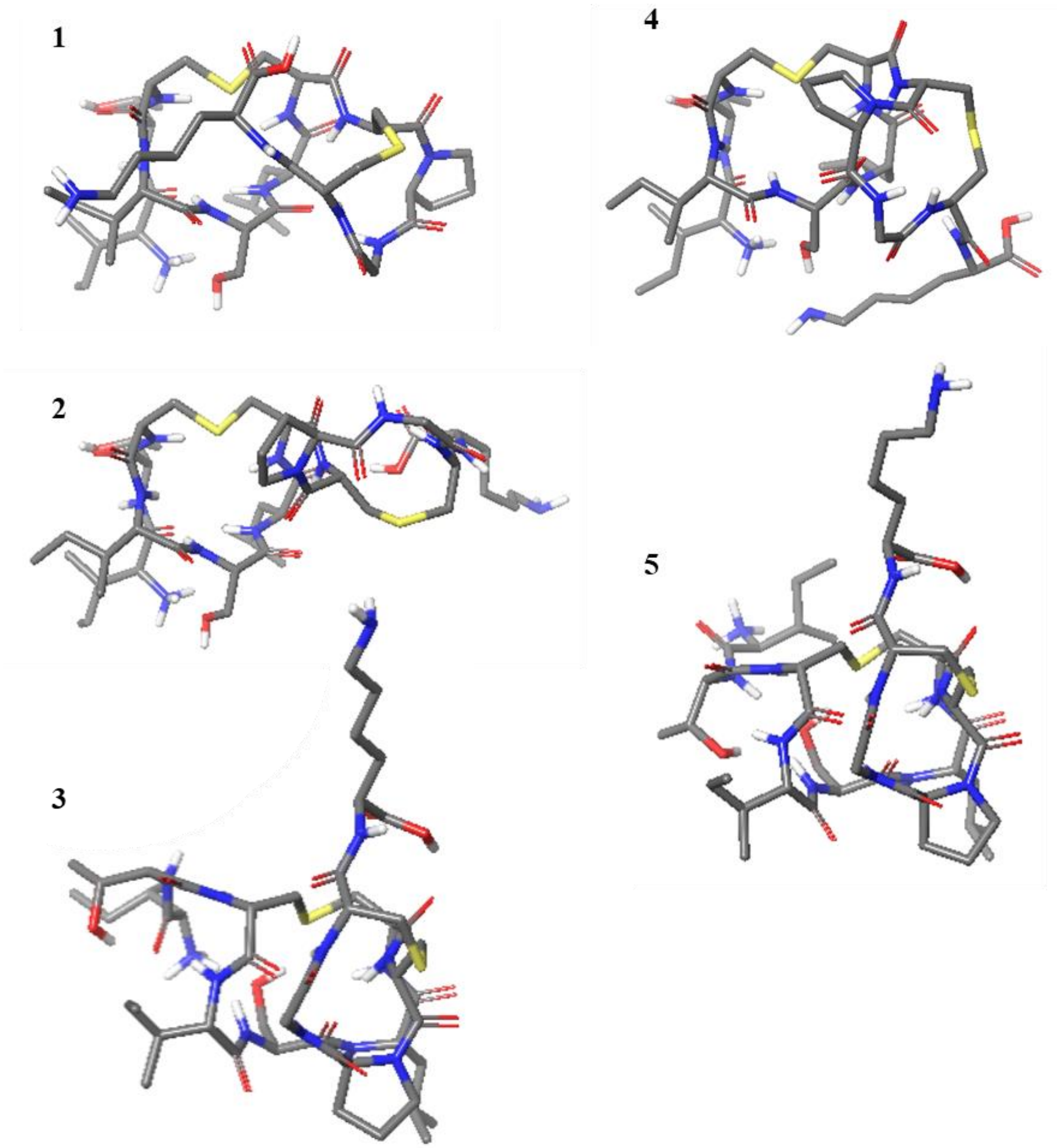

Figure S6: Comparison of all conformations in each NAMFIS solution. For each peptide, all conformations are compared to conformation 1, by alignment of the backbone within ring A. Conformations 5 and 6 of nisin(1-12) **1** adopt lipid II-binding conformations. All other conformations of nisin(1-12) and Thr/Ser analogue are not-lipid II-binding conformations. Non polar hydrogens and non-ring residues have been omitted for clarity

Nisin(1-12) **1** conformations aligned to conformation 1

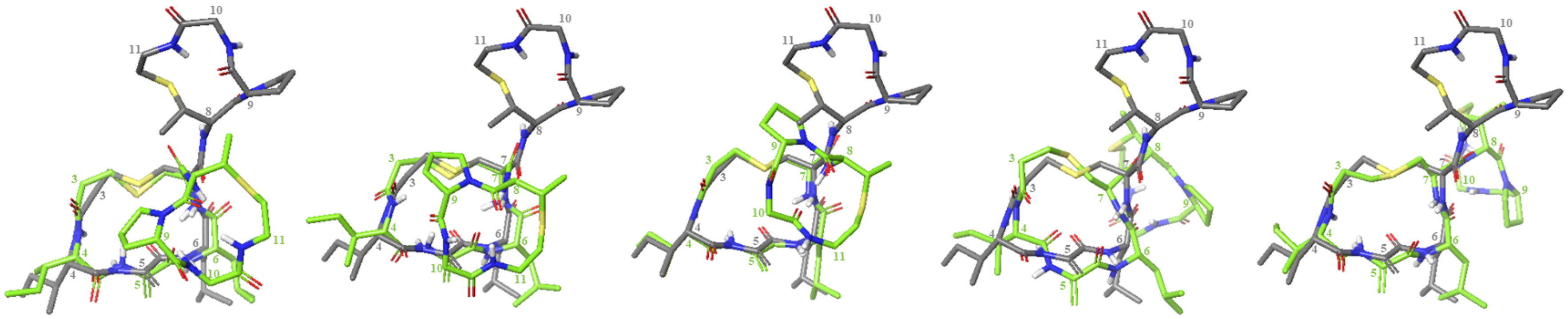

Conformation 2 Conformation 3 Conformation 4 Conformation 5 Conformation 6

(Thr2, Ser5) analogue **2** conformations aligned to conformation 1

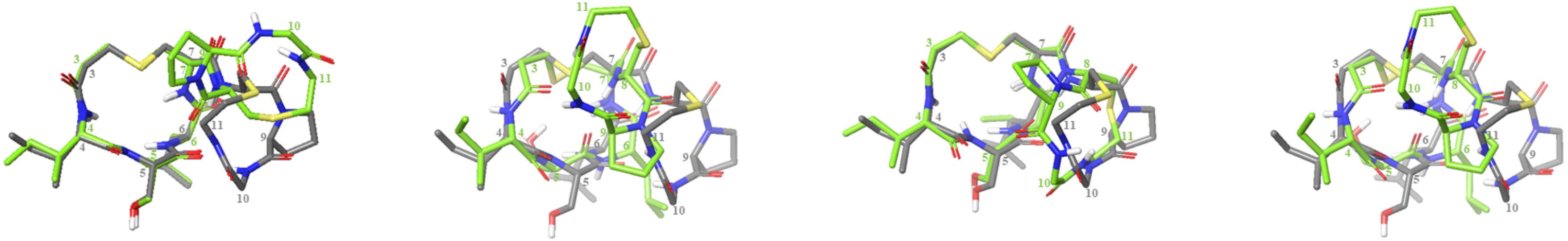

Conformation 2 Conformation 3 Conformation 4 Conformation 5

Figure S7: Comparison of the original (2*S*, 3*R*, 6*R*) and corrected (2*S*, 3*S*, 6*R*) structures of lipid II-bound nisin (PDB ID 1WCO). Non polar hydrogens have been omitted for clarity. Only the nisin(1-12) region is shown.

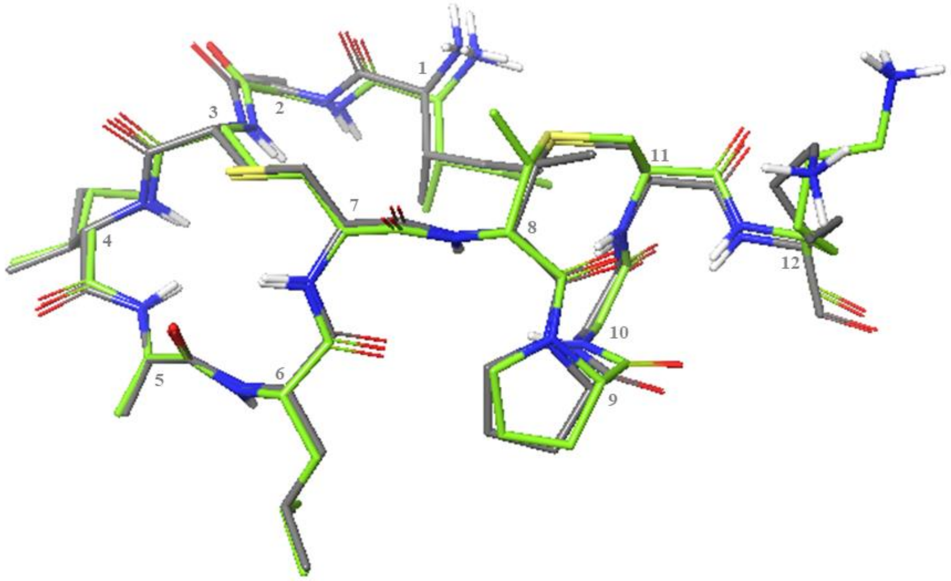

MeLan (2*S*, 3*R*, 6*R*)
MeLan (2*S*, 3*S*, 6*R*)

References

(1) R. Dickman, S. A. Mitchell, A. Figueiredo, D. F. Hansen, A. B. Tabor, *J. Org. Chem.* (submitted 2019).

(2) B. Mothia, A. N. Appleyard, S. Wadman, A. B. Tabor, *Org. Lett.* **2011**, *13*, 4216 – 4219.

(3) T. Koopmans, T. M. Wood, P. 't Hart, L. H. J. Kleijn, A. P. A. Hendrickx, R. J. L. Willems, E. Breukink, N. I. Martin, *J. Am. Chem. Soc.* **2015**, *137*, 9382 – 9389.

(4) J. C. Slootweg, R. M. J. Liskamp, D. T. S. Rijkers, *J. Pept. Sci.* **2013**, *19*, 692 – 699.

(5) N. Nevins, D. Cicero, J. P. Snyder, *J. Org. Chem.* **1999**, *64*, 3979 – 3986.

(6) S.-T. D. Hsu, E. Breukink, E. Tischenko, M. A. G. Lutters, B. de Kruijff, R. Kaptein, A. M. J. J. Bonvin, N. A. J. van Nuland, *Nat. Struct. Molec. Biol.* **2004**, *11*, 963 – 967.

(7) D. O. Cicero, G. Barbato, R. Bazzo, *J. Am. Chem. Soc.* **1995**, *117*, 1027 – 1033.

(8) V. F. Bystrov, *Prog. Nucl. Magn. Reson. Spectrosc.* **1976**, *10*, 41 – 82.
